# Molecular mechanisms in rare proteasomopathies

**DOI:** 10.1016/j.ero.2025.12.005

**Published:** 2026-01-17

**Authors:** Sophie Wolfgramm, Flavia Llorente Alvarez, Franziska G Thiel, Martin Wendlandt, Elke Krüger

**Affiliations:** Institute of Medical Biochemistry and Molecular Biology, University Medicine Greifswald, Greifswald, Germany

## Abstract

Proteasomopathies comprise rare interferonopathy-related syndromes caused by genetic defects in proteasomal subunits or their assembly factors resulting in failed proteasome biogenesis and/or function. The concomitant proteasome impairment leads to imbalanced protein homeostasis by dysfunctional ubiquitin-mediated protein degradation. Two distinct clinical phenotypes have been characterised in proteasomopathies so far: (i) proteasome-associated autoinflammatory syndromes and (ii) proteasome-associated neurodevelopmental disorders. Despite these differences, both syndromes show molecular similarities with protein aggregation, activated stress responses, metabolic imbalance and dysregulated type I interferon signalling. Diagnostics and clinical management are complex even if genetic information is available. Here, we integrate the current knowledge of mammalian proteasome biogenesis with structural modelling of known proteasomopathy-causing variants and discuss the innovations of structural modelling to accelerate diagnosis.

## PROTEASOME DYSFUNCTION IMBALANCES CELLULAR PROTEIN HOMEOSTASIS

The sensitive balance between protein synthesis and degradation determines protein homeostasis within eukaryotic cells. Along with lysosomal degradation mechanisms, the ubiquitin-proteasome system (UPS) governs the protein household [1]. Ubiquitin controls the fate of target proteins through its linkages and chain length [2]. This process is catalysed by a thioester cascade of E1, E2, and E3 enzymes to establish the isopeptide bond of ubiquitin at the substrate protein [3]. The attachment of lysine (K) K48-linked ubiquitin chains of at least 4 ubiquitin moieties signals degradation by the proteasome [4,5]. Deubiquitinating enzymes (DUBs) such as UCHL5/UCH38 and USP14 are associated or closely located to the proteasome to remove ubiquitin for degradation or to protect ubiquitin-labelled proteins from degradation, acting as negative regulator for proteasomal-mediated protein degradation [6,7]. By targeted proteolysis, the UPS is not only part of the proteostatic network in cells, but also controls numerous signalling, metabolic pathways, or genome stability. In immune responses, the proteasome generates antigenic or proteasome-derived defence peptides or determines the abundance of regulatory proteins [[8], [9], [10], [11], [12]].

**Figure 1:**
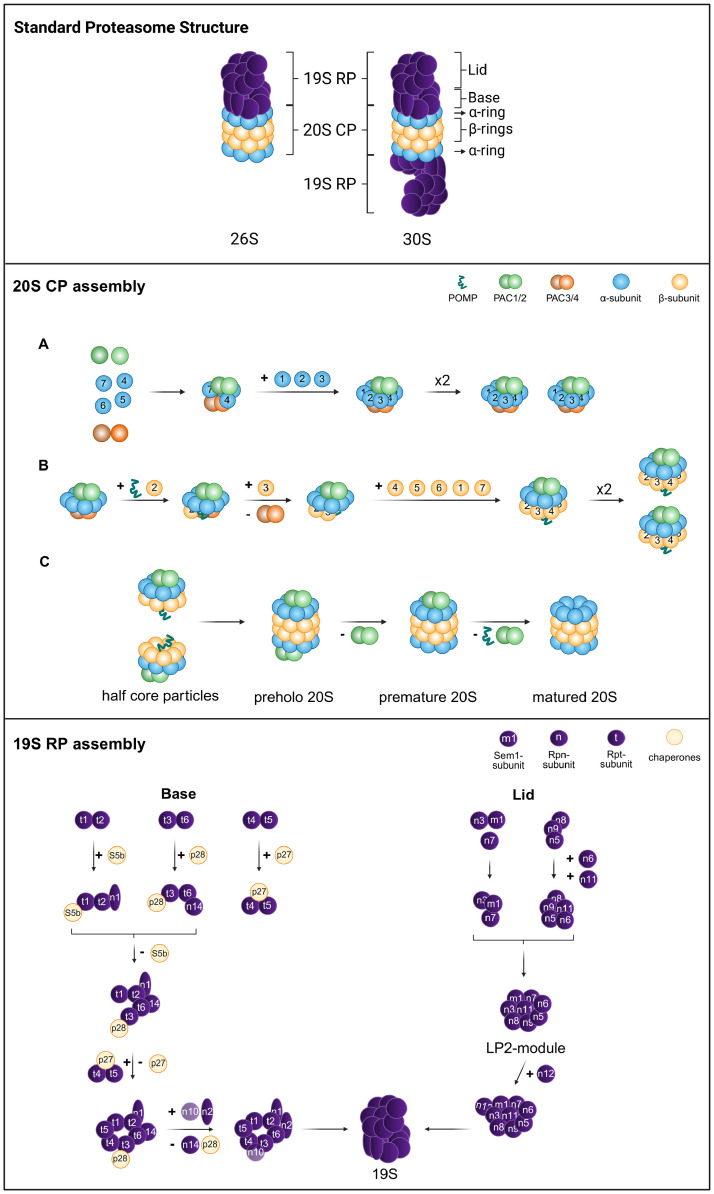
26S/30S standard proteasome structure and model mechanism of the standard 20S and 19S assembly in mammals. The 26S/30S standard proteasome is composed of a 20S proteolytic core particle capped by 1 or 2 19S regulatory particle(s). The 20S core particle encompasses 2 inner β-rings and 2 outer α-rings, following the order: α-β-β-α. The 19S regulatory particle, however, consists of the lid and base complex. 20S assembly: the 20S assembly is initiated by the formation of the α-ring facilitated by the chaperones PAC1/2 (green) and PAC3/4 (dark orange) (A). The α-ring serves as a platform for further β-subunit incorporation and thus half proteasome formation, promoted by the linker-protein POMP (dark green) (B). The fusion and the maturation of the proteasome through several steps (preholo 20S and premature 20S) finalises the 20S biogenesis (C). 19S assembly: the 3 modules, consisting of Rpt subunits, Rpn1 and the chaperones S5b, p28, p27, and Rpn14 facilitate base complex formation in the stepwise assembly process, from module fusion to Rpn2 incorporation. The association of Rpn10 (light purple) was not detected experimentally. According to the 19S lid assembly, 2 smaller subunit complexes build the LP2-complex. The Rpn12 involvement matures the lid, paving the association of the base. Purple circles with a ‘t’ or ‘n’ represent Rpt or Rpn subunits, respectively. Created in BioRender. Venz, S. (2026) https://BioRender.com/tocajkc. CP, core particle; RP, regulatory particle; PAC1/2, proteasome assembly chaperone 1/2; PAC3/4, proteasome assembly chaperone 3/4; POMP, proteasome maturation protein; Rpt, regulatory particle ATPase; Rpn, regulatory particle non-ATPase; LP2, lid subcomplex.

The standard 26S proteasome represents a prototypic ATP-dependent multicatalytic protease with a conserved architecture comprising the 20S catalytic core particle (CP) and the 19S regulatory particle (RP) (Fig 1) [13]. The 20S CP consists of stringently ordered heptameric α- and β-rings: α_7_-β_7_-β_7_-α_7_ [13,14]. Peptide-bond hydrolysis is associated with the 20S CP through 3 cleavage specificities of the 6 active site subunits conferring caspase-like activity assigned to β1, trypsin-like to β2 and chymotrypsin-like activity to β5, respectively [14,15]. On the contrary, the 19S RP consists of 2 main complexes, the base with a hexameric symmetry and the lid. The 19S RP governs ubiquitin binding, its cleavage from substrates, and its dissociation and translocation through ATP release [16]. Together, this multisubunit protease complex consists of 33 different subunits [17].

Proteasomes are formed by a sophisticated biogenesis program, which requires assembly helper proteins and that is conserved from yeast to humans [13].

Genetic alterations in proteasome-subunit or assembly helper genes have been associated with severe phenotypic manifestations, including a wide range of symptoms related to a failure in protein homeostasis. These rare diseases are summarised by the term: proteasomopathies, enclosing the historical description of several syndromes that share the same underlying cause and their relation to interferonopathies [[18], [19], [20]]. Two major phenotypes have been distinguished: proteasome-associated autoinflammatory syndrome (PRAAS) and proteasome-associated neurodevelopmental disorder (PRNDD), with each defined by its distinct clinical presentation [21]. Both PRAAS and PRNDD are linked to type I interferonopathies [18,22].

Diagnosis of proteasomopathies requires a complex procedure, covering clinical assessment, genetic profiling, as well as structural and functional analyses at the molecular level. A detailed unravelling of proteasome biogenesis turned out to enable the refinement of the proteasomopathy-associated molecular understanding. Key mechanistic steps in the proteasome assembly process together with the structural modelling tool AlphaFold3 [23] will boost the diagnostic procedure in rare proteasomopathies and will be further addressed in this review.

## HISTORICAL BACKGROUND OF PROTEASOMOPATHIES

**Figure 2:**
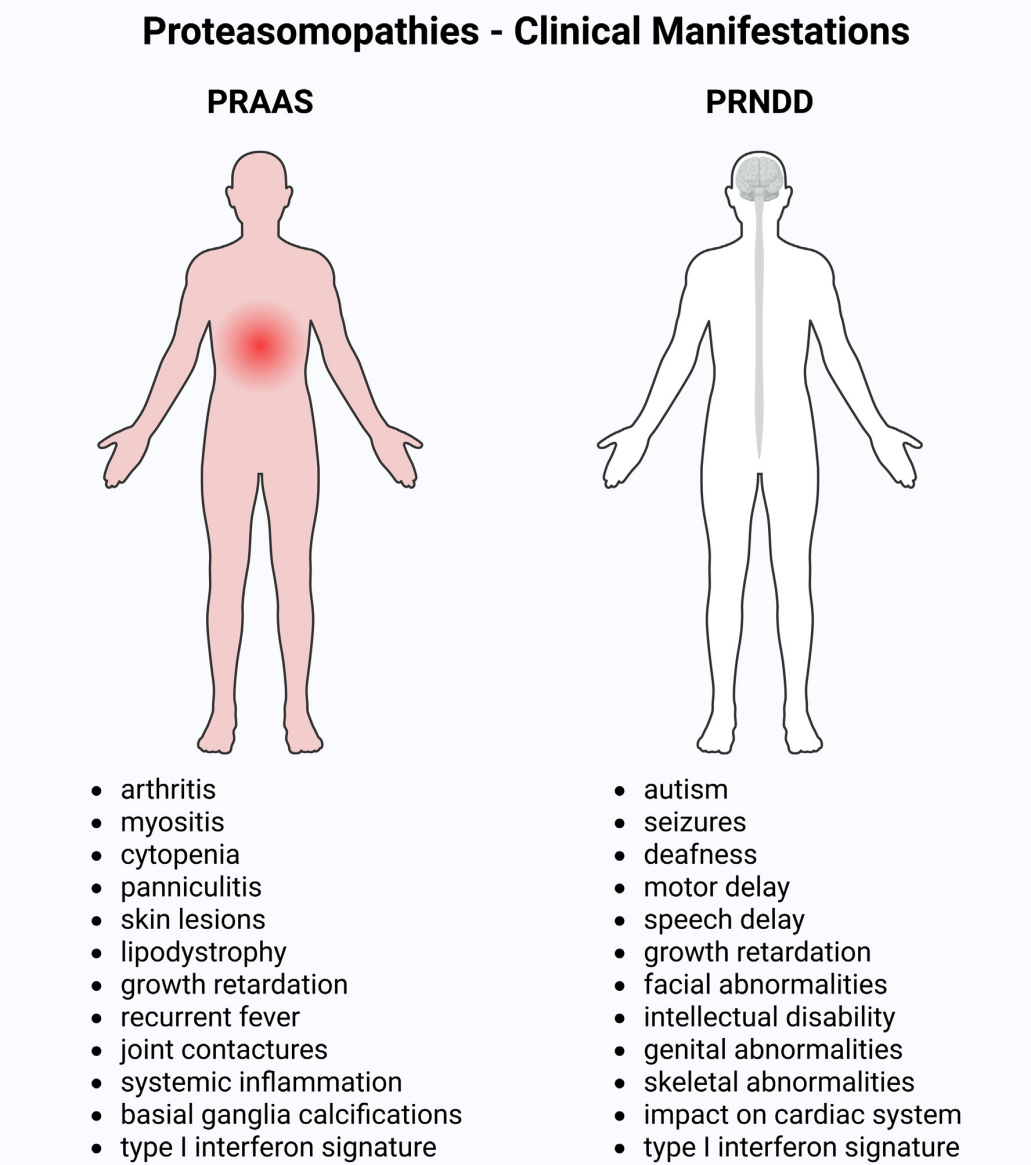
Principal clinical manifestations in PRAAS and PRNDD. Symptomatic spectrum of known patients with PRAAS and PRNDD. Not all reported symptoms of a given phenotype are present in every individual patient described in the literature. In some cases, overlaps in the clinical manifestations were observed. Created in BioRender. Venz, S. (2026) https://BioRender.com/d4ja9wz. PRAAS, proteasome-associated autoinflammatory syndromes; PRNDD, proteasome-associated neurodevelopmental disorders.

PRAAS were first described in the context of their clinical manifestations and later linked to genetic mutations affecting the proteasome. An early milestone came in the late 1930s, when Dr Nakajo reported a case of ‘secondary hypertrophic osteoperiostosis with pernio,’ which was later classified as Nakajo-Nishimura syndrome (NNS) [24]. In 2010, the first genetic analyses of NNS diagnosed patients revealed a pathogenic homozygous variant in the proteasomal subunit *PSMB8*, encoding β5i, a catalytic subunit of the immunoproteasome [25]. The main symptomatic features appear in the first months after birth and can develop over years, manifested by lipodystrophy, skin lesions, thickened fingers, growth retardation, recurrent fever and/or panniculitis (Fig 2) [11,25,[26], [27], [28], [29], [30], [31], [32], [33], [34], [35], [36], [37], [38], [39], [40], [41], [42], [43], [44], [45], [46], [47], [48], [49], [50], [51], [52], [53], [54]]. The broad spectrum of symptoms in PRAAS, accompanied by diverse clinical variations, has led to the classification of multiple syndromes, each one integrating key symptoms into its nomenclature: joint contractures, muscle atrophy, microcytic anaemia, and panniculitis-induced lipodystrophy; Japanese autoinflammatory syndrome with lipodystrophy; the chronic atypical neutrophilic dermatosis with lipodystrophy and elevated temperature, and the proteasome maturation protein (POMP)-related autoinflammation and immune dysregulation disorder [11,[25], [26], [27], [28], [29], [30], [31], [32], [33], [34],^36^,^37^,[39], [40], [41], [42], [43], [44], [45], [46], [47], [48], [49],^52^,^53^]. Novel cases of proteasome dysfunction, related to *PSMB10* mutations, were described as phenotypically related to severe combined immunodeficiency-Omenn-syndrome or immunodeficiency; however, the observed cytopenia in patients is not restricted to *PSMB10* deficiencies [35,50,54]. Still, 1 case carried 2 additional inherited variants in *PSMB2* and *PSME2* whose contribution to the disease remains unclear and needs further verification to be identified as truly disease-causing [50]. To date, therapeutic applications often target the suppression of the type I interferon-driven systemic inflammation through the use of JAK inhibitors, such as baricitinib or interferon receptor blocking antibodies (anifrolumab) in addition to classical anti-inflammatory drugs such as steroids [11,21,34,38,48,49,53,55]. In addition, successful haematopoietic stem cell transplantation has been documented in certain cases to alleviate symptoms [33,36,38].

Almost 80 years after the first documentation of PRAAS, PRNDD was characterised in 3 studies with loss-of-function mutations in the *PSMD12* gene encoding the 19S RP constituent Rpn5 [[56], [57], [58]]. Subsequent reports have documented additional cases within the *PSMD12* gene and other 19S subunit genes (*PSMC1, PSMC3, PSMC5*, and *PSMD11*), which were classified as Stankiewicz-Isidor syndrome or neurodevelopmental disorders (NDDs)/delay, respectively [12,[59], [60], [61], [62], [63], [64], [65], [66]]. These findings contributed to the hypothesis that the manifestation of the phenotype depends on the subcomplex. However, variants in the 20S subunits *PSMB1* and *PSMA7* challenged this hypothesis as they caused cases of classified PRNDD and severe intellectual disability, respectively [67,68]. Common symptoms include neurodevelopmental delay, speech delay, intellectual disability, impaired sensing functions, hypotonia, general developmental delay, and in some cases seizures (Fig 2) [12,[56], [57], [58], [59], [60], [61], [62], [63], [64], [65], [66], [67]]. Although distinct clinical manifestations were observed for PRAAS and PRNDD, symptomatic overlaps between the 2 phenotypes in several cases exist such as intellectual disability, growth retardation, recurrent infections, thrombocytopenia, and periodic fever [12,55]. Even proteasome-associated proteins, E3 ligases, and deubiquitinating enzymes such as USP14 [7], USP7 [69], USP9X [70,71], UBE3A [72,73], UBE3C [74], and HUWE1 [75] exhibit neurodevelopmental and intellectual disability manifestations, which are overlapping with the PRNDD spectrum. Detailed clinical and molecular characteristics of proteasome-related NDDs were summarised by Cuinat et al [19].

## MECHANISM OF MAMMALIAN PROTEASOME BIOGENESIS

Our current understanding of proteasome biogenesis is limited and often based on analyses in yeast. Mammalian proteasome biogenesis is more sophisticated due to proteasome heterogeneity with a couple of isoforms due to alternative subunits or regulator complexes (see below).

The nomenclature of proteasome subunits is puzzling, because the rationale of gene names does not fit to the rationale of subunits in resolved structures (eg, *PSMB6* encodes β1; for details, Table 1). The yeast nomenclature for 19S RP is commonly used for resolved structures of mammalian-based 26S proteasomes. Therefore, the description of the 19S RP biogenesis and involved subunits refer in this review to the yeast nomenclature.

**Table 1:** List of proteasomal subunits and proteasome-associated subunits according to the UniProt database accession numbers and annotated functions, including a list of resolved proteasome structures and their related PDB identifiers.

19S regulatory particle (RP)				
Protein	Gene	Accession no.	Additional information	
Rpn1	*PSMD2*	Q13200	Non-AAA ATPase	Possible binding to TNFR1
Rpn2	*PSMD1*	Q99460		
Rpn3	*PSMD3*	O43242		
Rpn5	*PSMD12*	O00232		
Rpn6	*PSMD11*	O00231		Important for 26S assembly
Rpn7	*PSMD6*	Q15008		
Rpn8	*PSMD7*	P51665		
Rpn9	*PSMD13*	Q9UNM6		
Rpn10	*PSMD4*	P55036		Recognises ubiquitin
Rpn11	*PSMD14*	O00487		Metalloprotease
Rpn12	*PSMD8*	P48556		
Rpn13	*ADRM1*	Q16186		Activates 19S-deubiquitinase (Rpn11)
SEM1/DSS1	*SEM1*	P60896		Possibly part of TREX-2 complex, binds BRCA2
Rpt1	*PSMC2*	P35998	AAA ATPase	Unfolds ubiquitinated proteins
Rpt2	*PSMC1*	P62191		
Rpt3	*PSMC4*	P43686		
Rpt4	*PSMC6*	P62333		
Rpt5	*PSMC3*	P17980		
Rpt6	*PSMC5*	P62195		

20S catalytical core particle (CP)				

α1	*PSMA6*	P60900	α-ring	Association with regulators and activators
α2	*PSMA2*	P25787		
α3	*PSMA4*	P25789		
α4	*PSMA7*	O14818		
α4s	*PSMA8*	Q8TAA3		Spermatoproteasome-specific
α5	*PSMA5*	P28066		Association with regulators and activators
α6	*PSMA1*	P25786		
α7	*PSMA3*	P25788		
β1	*PSMB6*	P28072	β-ring	Active site, caspase-like activity
β2	*PSMB7*	Q99436		Active site, trypsin-like activity
β3	*PSMB3*	P49720		
β4	*PSMB2*	P49721		
β5	*PSMB5*	P28074		Active site, chymotrypsin-like activity
β6	*PSMB1*	P20618		
β7	*PSMB4*	P28070		Mediates degradation in complex with SMAD1/OAZ1 of SNIP1
β1i	*PSMB9*	P28065		Immunoproteasome-specific, active site, caspase-like activity
β2i	*PSMB10*	P40306		Immunoproteasome-specific, active site, trypsin-like activity
β5i	*PSMB8*	P28062		Immunoproteasome-specific, active site, chymotrypsin-like activity
β5t	*PSMB11*	A5LHX3		Thymoproteasome-specific, active site

Assembly helpers				

Rpn14	*PAAF1*	Q9BRP4	19S assembly helper	
S5b	*PSMD5*	Q16401		
p27	*PSMD9*	O00233		
p28	*PSMD10*	O75832		Known as proto-oncoprotein
PAC1	*PSMG1*	O95456	20S assembly helper	Bind α-subunits during 20S assembly
PAC2	*PSMG2*	Q969U7		
PAC3	*PSMG3*	Q9BT73		
PAC4	*PSMG4*	Q5JS54		
POMP	*POMP*	Q9Y244		Linker for 20S assembly

Proteasome activators and inhibitor				

PA28α	*PSME1*	Q06323		Involved in antigen processing
PA28β	*PSME2*	Q9UL46		
PA28γ	*PSME3*	P61289		Enhances trypsin-like activity in proteasome
PA200	*PSME4*	Q14997		Part of ATP- and ubiquitin-independent protein degradation, recognition of acetylated histones
PI31	*PSMF1*	Q92530		Influences the proteasome activity

Resolved structures				

Type		PDB	Reference	
20S standard		8QYO, 7NAN, 7PG9, 6RGQ, 6R70, 5LE5, 4R3O	[[76], [77], [78], [79], [80], [81], [82]]	
20S standard preholo-proteasome (preholo-1)		8QYS, 8YIY, 8TM6,	[76,83,84]	
20S standard preholo-2		8YIZ	[84]	
20S immunoproteasome		7B12, 7AWE, 7DR7, 6AVO, 6E5B, 3UNH	[[85], [86], [87], [88], [89], [90]]	
26S standard		8CVT	[81]	
30S standard		5GJR	[91]	
PA28αβ+20S		8CXB, 7NAO	[81]	
PA28αβ+20S+ PA28αβ		7NAP	[81]	
PA28αβ+20S immuno		7DR6	[88]	
PA28αβ+20S immuno+ PA28αβ		7DRW	[88]	
PA200+20S		8CVS, 7NAQ, 6KWY	[81,92]	
PA200+20S+PA200		6REY	[82]	

AAA, ATPases associated with diverse cellular activities; TREX-2, transcription and export complex 2; BRCA2, breast cancer type 2 susceptibility protein; TNFR1, tumor necrosis factor receptor-1; SMAD1/OAZ1, suppressor of mothers against decapentaplegic homolog 1/ornithine decarboxylase antizyme 1; SNIP1, smad nuclear-interacting protein 1.

## 20S STANDARD PROTEASOME ASSEMBLY

The standard 20S structure and its finely tuned chaperone-guided biogenesis involving the initial formation of α-rings ensuring coordinated β-subunit incorporations is conserved from yeast to mammals [13,[93], [94], [95], [96]].

The assembly of the standard 20S CP in mammals is orchestrated by the assembly helper proteins PAC1-4 and POMP (Table 1, Fig 1) [94,83,[97], [98], [99], [100], [101], [102], [103]]. Precursor complexes, including the oppositely orientated heterodimers PAC1/2 and PAC3/4 on the α-subunits 4-7, have been reported with distinct interaction partners [83,99,100,102,103]. Both heterodimers showed an association with α5 and α6, strengthened by PAC1 and the formed α5/α6-HbYX binding pocket, whereas PAC2 and PAC3 are additionally linked to α7 [83]. The step-by-step assembly process of the remaining α-subunits, specifically α1-3, has not yet been fully clarified. To date, it is known that before α1 incorporation is the limitation for α2 association [83,103]. In regard to the ongoing assembly process, the N-terminal tails from α2-4 protect the gate to shield an early protein entrance [83].

Correct β-ring assembly strictly depends on POMP. Its recruitment facilitates β2 entry, followed by β3, β4, β5, β6 onto the α-ring [76,83,95]. The β3 incorporation forces the dissociation of PAC3 from α7 [76,83,95]. The β1 subunit is critical for the final completion of the β-ring via β7, which is alleviated by the propeptides of β1 and β5 [84,95]. These bottlenecks confirm previous observations of different intermediate states in proteasome assembly—the 13S and 16S [95,104,105]. After dimerisation of 2 half proteasomes, a loosely structured preholo-proteasome is formed [76]. A gradual process initiates propeptide cleavage for proteasome maturation [76,84]. Propeptide cleavage of β5 may occur first, followed by β1 and β2, as suggested by conformational changes in the catalytic pockets [83,84]. POMP cleavage and its subsequent degradation are likely performed by β2 and finalised through β5, forming the premature 20S proteasome [76,84]. These initiated conformational changes force PAC1/2 release and finalise the maturation of the 20S CP [76,83,84].

An accurate autocatalytic process is indispensable for proper proteasome biogenesis and activation of the active site threonines. The propeptides of the β-subunits (except β3 and β4) ensure successful ring formation and prevent premature activity or inactivation of the active sites through acetylation [[106], [107], [108], [109]]. Following the discovery of the propeptide-dependent inactivity of β-subunits and the 3 active cleavage site specificities, an additional restriction by the conserved glycine residue at position -1, relative to the catalytic threonine for the autocatalytic cleavage, was identified [14,15,[110], [111], [112]]. A 2-step mechanism has been proposed to explain the *trans*-functional autocatalytic cleavage of the propeptides, orchestrated by Thr, Lys, Ser, Asp, and a water molecule [106,[113], [114], [115]]. A recent study in yeast has even suggested a catalytic pentad mechanism involving the conserved pairs Ser/Asp and Lys/Asp next to the active threonine [116].

## 19S ASSEMBLY—ONE REGULATOR OF THE PROTEASOME

The 19S RP requires 2 independent multistep assembly processes for the base and lid subcomplex, respectively [117]. The base contains 6 AAA-ATPases (Rpt1-6) arranged in a hexameric ring configuration and 4 non-AAA-ATPases (Rpn1, 2, 13 and 10), while the lid exclusively harbours non-AAA-ATPases (Rpn) [13,91] (Fig 1).

Formation of ATPase-pairs (Rpt1-Rpt2, Rpt3-Rpt6, and Rpt4-Rpt5) of the base subcomplex is a prerequisite for module building in 19S regulator assembly (Fig 1) [[118], [119], [120]]. Three modules with their distinct chaperones, S5b, p27, or p28, are formed: S5b-Rpn1-Rpt1-Rpt2 module; p27-Rpt5-Rpt4 module; p28-Rpt3-Rpt6-Rpn14 module [118,121,122]. The p28 and S5b modules fuse to a larger complex, allowing the p27 module to join the precursor complex. Finally, Rpn2 can be incorporated into the complex and is located in close proximity to Rpt6 [118,123]. Rpn10 is not directly involved in base assembly; however, it stabilises the base lid complex together with Rpn1 and Rpn2 [118,120,123].

A comprehensive analysis by Bai et al [[124], [125], [126]] provides a detailed picture of the entire 19S lid assembly in mammals, which is similar in yeast. Two complexes are formed before their fusion to the LP2-module. Rpn3 and Rpn15 dimerisation triggers the association of Rpn7, yielding a tricomplex. Another module is initiated by the interaction between Rpn5, Rpn8, and Rpn9, and further incorporation of Rpn6 promotes association with Rpn11. The LP2-module is formed by the interaction between Rpn6 and Rpn7, followed by the incorporation of Rpn12 to complete the maturation of the lid [124]. To date, the participation of SEM1/DSS1 within the 19S RP lid biogenesis for mammalian cells has not been experimentally shown. Regarding the general conserved biogenesis in eukaryotic cells, its involvement in the lid biogenesis is likely essential [127].

The final steps of 26S proteasome formation associating 20S CP with 19S RP remain less well defined. Clearly, the HbYX binding motifs of several Rpt subunits in the 19S base support interaction with the α-ring and are responsible for gate opening to enable substrate insertion [16,128]. Previous studies in mammalian models propose that the 19S base chaperone p28 may act as a guardian of 26S proteasome assembly [128,129]. Additionally, Rpn14 encoded by PAAF1 is also required for proper 26S assembly, which shifts the role of 19S base chaperones into a key position during 26S formation [[128], [129], [130]]. Adaptor proteins such as Ecm29/ECPAS have been demonstrated to stabilise the proteasome [131].

## PROTEASOME HETEROGENEITY

Mechanistic biogenesis studies are mainly restricted to standard proteasomes. However, a couple of alternative subunits, activator and inhibitor complexes exist in mammals, adding to proteasome heterogeneity. Therefore, various proteasome isoforms can be generated, each with different degradation capacity or peptide hydrolysis activity often expressed in a tissue-specific manner. The synergy of proteasome isoforms with alternative subunits, regulators, activators, and inhibitor is thought to adapt proteolytic requirements to the environmental conditions and/or tissue or cell specificity; however, the exact function of these isoforms or their spatiotemporal regulation often remains obscure.

PA28αβ preferentially associates with immunoproteasomes and accelerates their peptide-hydrolytic capacity in a ubiquitin-independent manner thereby improving antigenic peptide generation during infection or inflammation [132]. By contrast, PA28γ enhances the basic amino acid cleavage and shapes the proteasome-derived defence peptide generation [10]. Interestingly, the role of PA200 still remains elusive and is typically linked to male fertility and cancer [133,134]. The proteasome inhibitor PI31 is known to block proteasome activity [135]. Interestingly, recent studies suggest an important role of PI31 during immune proteasome biogenesis [136,137]. In addition, a neuroprotective role of PI31 was observed and highlights its role in neurodegeneration [138,139].

**Figure 3:**
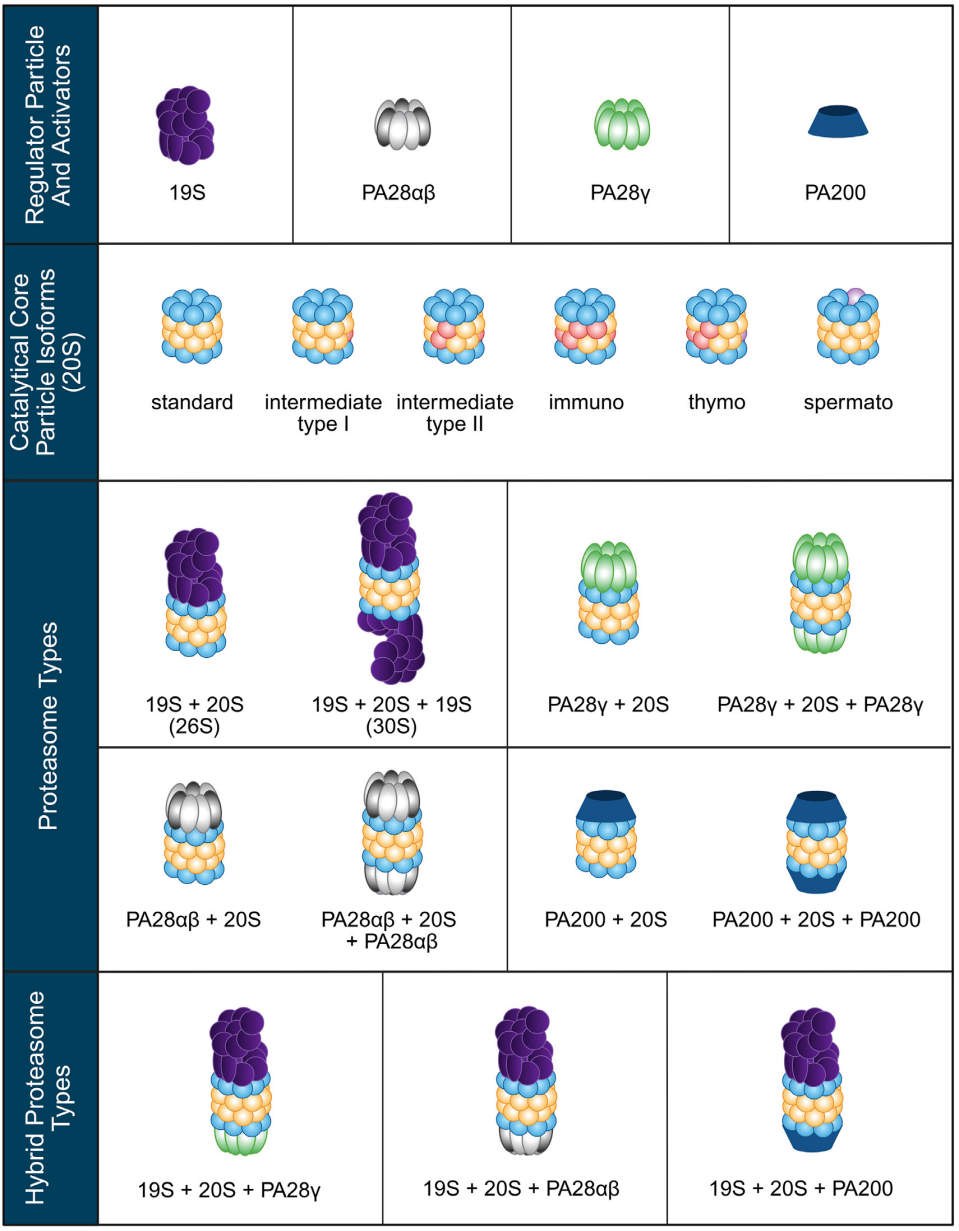
Heterogeneity of the catalytic core particle and proteasome (hybrid) types in mammals. The overview visualises the complexity of proteasome isoform biogenesis and the interplay of regulatory proteins in maintaining proteasome dynamics. Created in BioRender. Venz, S. (2025) https://BioRender.com/zzp092w.

Incorporation of alternative subunits results in different proteasome isoforms namely: intermediate proteasome, immunoproteasome, thymoproteasome and spermatoproteasome. The modular design of proteasome structures with the attachment of different regulators or the incorporation of alternative subunits allows a variety of possible proteasome types including hybrid forms (Fig 3) [8,140].

The so-called immunosubunits β1i, β2i, or β5i are constitutively expressed in immune cells or induced by type I or type II interferons. Their incorporation generates intermediate or immunoproteasomes [141]. Two types of intermediate proteasomes have been identified in different tissues with the exchange of β5 to β5i, or incorporation β1i, and β5i [[142], [143], [144]]; however, their exact function requires better experimental proof.

Due to the genetic locus of *PSMB8* and *PSMB9* (encoding β1i and β5i) within the major histocompatibility complex (MHC) region, immunoproteasome function was initially exclusively associated with MHC class I antigen presentation [145]. However, additional immunoproteasome functions are outlined in the comprehensive review from Ebstein et al [146]. Its active site subunits β1i, β2i, and β5i increase the catalytic activity along with favoured cleavage of basic and hydrophobic amino acid residues to confer adaptation to increased requirements for improved proteostatic potential under conditions such as haematopoietic differentiation. A different β-subunit assembly hierarchy, combined with a preferential POMP-β5i interaction, could explain the more rapid formation kinetics of immunoproteasomes [147,148].

The nonessential incorporation of β4 before β5i is contrary to its β5 counterpart in the standard proteasome biogenesis [147]. The final step for half-immunoproteasome assembly is the incorporation of β6 and β7. The early recruitment of β1i and β2i may be important for the faster assembly of the immunoproteasome dependent on their propeptides [141]. A model suggested the critical incorporation of β1i for β2i, irrespective of β5i [141]. Nevertheless, β5i contributes to a faster β1i and β2i processing as its propeptide plays a major role in the interaction with β1i [141,149].

The thymus matures naive T lymphocytes by peptide antigenic generation through another special proteasome subtype, the thymoproteasome [8,140,150]. The interplay between the thymoproteasome and the immunoproteasome enables proper positive and negative selection, due to their localisation to cortical thymic epithelia cells and dendritic cells, and medullary thymic epithelial cells, respectively [[151], [152], [153], [154], [155], [156], [157], [158]]. In addition to β1i and β2i, the thymoproteasome possesses a distinct β5t subunit [152,156]. A similar assembly process as that of the immunoproteasome has been proposed, suggesting a favoured incorporation of β5t in the presence of IFN-γ as shown in *in vitro* studies using mouse embryonic fibroblasts [147]. By generation of a peptide repertoire different from that of the immunoproteasome due to a decrease of the chymotrypsin-like activity, the thymoproteasome has been shown to support positive selection [147,152,153,156,159].

The spermatoproteasome, in turn, is specifically expressed in the testis and is involved in spermatogenesis by replacing the α4 subunit with α4s in the 20S, containing standard proteasomal active sites [160,161]. The spermatoproteasome is often associated with the regulator PA200 [92], which is essential for fertility and possibly responsible for the histone degradation in an acetylation-dependent manner [133].

## DIAGNOSTIC PROCEDURES IN PROTEASOMOPATHIES

Genetic assessment of proteasomopathies turned out to be a challenge due to 52 genes encoding standard or alternative proteasome subunits, regulator subunits or proteasome assembly helpers (Table 1). In addition, there is inconsistent nomenclature due to numbering of genes different from subunits within the structure. Loss-of-function (LOF) variants in proteasomopathies span a broad spectrum of regulator- or isoform-related subunits, adding to the molecular complexity of these diseases. Of the 52 known proteasome or assembly helper genes, 17 are currently known to be affected in proteasomopathies resulting in this diverse spectrum of different syndromes, although they all impact 1 multisubunit enzyme complex, the proteasome (Table 2).

**Table 2:** List of published variants in proteasomal subunits (26S) and proteasome-associated assembly helpers

PRAAS and SCID								
	Gene (transcript)	HGVS	Experimental outcome				Additional information	Ref
			Genetic/structural prediction^a^	Cell model				
				Proteasome biogenesis	Proteasome activity/function	Affected protein maturation		
20S (Standard)	*PSMA3* (NM_002788.4)	c.404+2T>C p.(His111Phefs*10)	ND	ND	ND	ND	Unstable transcript	[30]
		c.696_698del p.(Arg233del)	Deleterious	HeLa cells: 20S incorporation, very less content (V5)	ND	HeLa cells: no matured form		[30]
	*PSMA5* (NM_002790.4)	c.502C>T p.(Arg168*)	Structural modelling: possibly disruption of interaction with Rpt1	HeLa cells: no incorporation (V5)	ND	HeLa cells: no expression		[39]
	*PSMA6* (NM_002791.3)	c.171+64_253+528del	Classification: probably pathogenic	ND	ND	ND		[52]
	*PSMB2* (NM_002794.5)	c.142G>A p.(Gly48Arg)	ND	ND	ND	ND		[50]
	*PSMB4* (NM_002796.3)	c.-9G>A p.?	Reduced splicing prediction	HeLa cells: normal incorporation, less content (V5)	ND	HeLa cells: truncated, less content		[30]
		c.44dup p.(Pro16Serfs*45)	ND	ND	ND	ND	Published as c.44_45insG	[30]
		c.231del p.(Leu78Trpfs*31)	Pathogenic	ND	ND	ND		[36]
		c.494+17A>G p.?	ND	ND	ND	ND		[36]
		c.636_644del p.(Asp212_Val214del)	Deleterious	HeLa cells: normal 20S incorporation with less content, presence of precursor and intermediate form, absent 26S incorporation (V5)	ND	HeLa cells: absent maturation, higher proform content	Published as c.634-642del	[30]
		c.666C>A p.(Tyr222*)	ND	HeLa cells: normal 20S incorporation with very less content, presence of precursor and intermediate form, absent 26S incorporation (V5)	ND	HeLa cells: truncated protein		[30]
20S (Immuno)	*PSMB8* (NM_148919.4)	c.163C>T p.(Gln55*)	ND	HeLa cells: absent incorporation	ND	HeLa cells: absent expression		[39,48]
		c.224C>T p.(Thr75Met)	Probably damaging/damaging, deleterious; structural modelling: possible impact on proteolytic activity	HeLa cells: normal incorporation, less 20S/26S content, higher presence of 16S (V5)	ND	HeLa cells: normal, less content		[25,26,30,39,40,52,53]
		c.269C>T p.(Ser90Phe)	Deleterious; structural modelling: close to active site pocket S1 and cleavage site of propeptide	ND	ND	ND		[51]
		c.274G>A p.(Ala92Thr)	Probably damaging/damaging, deleterious	HeLa cells: normal incorporation (V5)	ND	HeLa cells: normal		[26,28,30,45]
		c.275C>T p.(Ala92Val)	ND	ND	ND	ND		[34]
		c.280G>C p.(Ala94Pro)	Probably damaging/damaging, deleterious	ND	ND	ND		[32]
		c.313A>C p.(Lys105Gln)	Probably damaging/ damaging, deleterious	HeLa cells: normal incorporation (V5)	ND	HeLa cells: absent maturation, presence of proform and intermediate form		[30,34]
		c.349A>G p.(Met117Val)	Probably damaging/damaging, deleterious	HeLa cells: normal incorporation, less intermediate and 20S+PA28 content (V5)	ND	HeLa cells: normal		[30,46]
		c.352T>C p.(Ser118Pro)	Structural modelling: possible impact on the secondary structure	HeLa cells: normal 16S/20S incorporation with less content, absence 26S (V5)	ND	HeLa cells: normal, very less content for processed form		[39,48]
		c.367G>A p.(Asp123Asn)	Damaging	ND	ND	ND	Published as NM_004159.5:c.355G>A p.(Asp119Asn)	[27]
		c.385C>T p.(Arg129Cys)	Damaging	ND	ND	ND	Published as NM_004159.5:c.373C>T p.(Arg125Cys)	[27]
		c.389del p.(Leu130Argfs*29)	ND	ND	ND	ND	Published as p.129Argfs*27	[47]
		c.405C>A p.(Cys135*)	ND	HeLa cells: absent incorporation (V5)	ND	HeLa cells: very less to absent expression, presence of truncated form		[30,40]
		c.602G>T p.(Gly201Val)	Structural modelling: possible clash between β5i and β4	HeLa cells: normal 20S incorporation with very less content, intermediate accumulation (V5)	ND	HeLa cells: absent maturation, presence of proform and intermediate form	Published as NM_004159.5: c.590G>T p.(Gly197Val)	[29,30,42,47]
		c.625G>A p.(Gly209Arg)	Classification: likely pathogenic; structural modelling: possible clash between β5i and β4; classification: likely pathogenic	HeLa, EA.hy.926, and C20: stalled incorporation, probably at premature state (V5)	HeLa: slightly reduced in chymotrypsin-like activity EA.hy.926, and C20: normal chymotrypsin-like activity	HeLa, EA.hy.926, and C20: absent maturation		[11]
		c.625G>C p.(Gly209Arg)						
		c.704C>A p.(Ala235Asp)	Deleterious; structural modelling: located close at β5i/β4 interface, possibly steric clash	ND	ND	ND		[51]
	*PSMB9* (NM_002800.5)	c.467G>A p.(Gly156Asp)	Structural modelling: possible steric clash (20S)	Mouse model: β1i incorporation into matured proteasome impaired, presence of intermediate form	Mouse model: slightly reduced chymotrypsin-like activity	ND		[31,44]
		c.494G>A p.(Gly165Asp)	Benign, deleterious, tolerated	ND	ND	ND	To unstable for expression	[30]
	*PSMB10* (NM_002801.4)	c.40_42del p.(Phe14del)	Structural modelling: located in propeptide sequence, possible influence in proteasome biogenesis	HeLa cells: normal 16S/20S incorporation, highly reduced 20S content, highly increased 16S content, absent 26S incorporation (V5)	ND	HeLa cells: absent maturation, presence of proform and intermediate form		[39]
		c.41T>C p.(Phe14Ser)	Probably damaging/damaging, deleterious	ND	HEK293T cells: reduced trypsin-like and chymotrypsin-like activity	HeLa cells: absent maturation, presence of proforms		[43]
		c.166G>C p.(Asp56His)	Deleterious	ND	ND	ND		[35]
		c.247dup p.(Cys83Leufs*123)	ND	ND	ND	ND	Published as c.247_248insT, possibly NMD	[39]
		c.500G>A p.(Gly167Asp)	Structural modelling: located in ninth β-sheet	HeLa cells: normal 16S/20S incorporation with less content, absent 26S incorporation (V5)	ND	HeLa cells: absent maturation, presence of proform		[39]
		c.601G>A p.(Gly201Arg)	Deleterious, likely pathogenic	ND	ND	HEK293T cells: upper shift indicating intermediate form; HeLa cells: absent maturation, presence of proform		[35,50]
		c.601G>C p.(Gly201Arg)	Deleterious	ND	ND	ND		[35]
		c.614A>C p.(Asp205Ala)	ND	HEK293T cells: normal incorporation into assembly intermediate, no incorporation into 26S/30S proteasome (V5)	ND	HEK293T cells: maturation, but very less content; presence of proform (V5)		[54]
		c.623C>T p.(Ser208Phe)	ND	HEK293T cells: normal incorporation into assembly intermediate, no incorporation into 26S/30S proteasome (V5)	ND	HEK293T cells: maturation, but very less content; presence of proform (V5)		[54]
		c.710 + 1G>C p.?	Structural modelling: predicted exon 7 loss, possibly impact on proteasome biogenesis	ND	ND	ND		[39]
Assembly helpers (20S)	*POMP* (NM_015932.6)	c.326dup p.(Asp109Glufs*2)	ND	ND	ND	ND		[33]
		c.334_335del p.(Ile112Trpfs*3)	ND	HEK293T cells: presence of precursor (α6)	ND	HEK293T cells: normal, highly less content, strong presence of truncated version	Published as c.333_334del p.(Ile112Trpfs*3)	[37]
		c.340_344dup p.(Glu115Aspfs*20)	ND	HeLa siRNA model: less content (α6)	HeLa siRNA model: highly reduced chymotrypsin-like activity	HeLa siRNA model: slight expression	Published as c.344_345insTTTGA; to unstable for expression	[30]
		c.342_348delinsACC p.(Phe114Leufs*18)	ND	HEK293T cells: presence of precursor (α6)	ND	HEK293T cells: normal, highly less content, strong presence of truncated version		[37]
	*PSMG2* (NM_020232.5)	c.666_667del p.(Tyr223Serfs*2)	ND	HeLa cells: reduced incorporation (FLAG)	ND	HeLa cells: truncated protein		[41]
		c.675T>G p.(Asn225Lys)	ND		ND	HeLa cells: normal, less content		[41]
19S	*PSMC5* (NM_002805.6)	c.1080+1_1080+10del p.(Ala324_Lys360)	Structural modelling: possibly tolerated	HeLa cells: incorporation into precursor and intermediate forms, absent in 26S (HA)	ND	HeLa cells: truncated protein	Detected exon 10 skipping resulting in in-frame deletion; exon 10 is in the AAA ATPase domain	[39]
PA28β	*PSME2* (NM_002818.3)	c.512G>A p.(Arg171His)	ND	ND	ND	ND		[50]


PRNDD								
	Gene (transcript)	HGVS	Experimental outcome				Additional information	Ref
			Genetic/structural prediction^a^	Cell model				
				Proteasome biogenesis	Proteasome activity/function	Affected protein maturation		
20S (Standard)	*PSMB1* (NM_002793.4)	c.307T>C p.(Tyr103His)	Possibly partial loss-of-function; structural modelling: possible influence on interaction between β6 and α5	SHSY5Y cells: normal incorporation (α6, myc), less overall content (myc)	SHSY5Y cells: slightly reduced chymotrypsin-like activity (48h)	SHSY5Y cells: maturation absent, proform present (myc)		[67]
19S	*PSMC1* (NM_002802.3)	c.983T>C p.(Ile328Thr)	Damaging and possibly damaging; structural modelling: located at AAA-ATPase region, possibly impact on protein’s hydrophobic core	ND	ND	ND		[62]
	*PSMC3* (NM_002804.5)	c.511C>T p.(Arg171Trp)	Intolerant; structural modelling: disrupted polar network building	ND	ND	SHSY5Y cells: no expression		[61]
		c.523A>G p.(Met175Val)	Pathogenic; structural modelling: possibly destabilisation of tertiary structure	SHSY5Y cells: normal incorporation into 19S butless content, 26S incorporation absent (V5)	ND	SHSY5Y cells: normal, slightly less content		[61]
		c.686C>T p.(Pro229Leu)	Intolerant	ND	ND	ND		[61]
		c.710C>T p.(Ala237Val)	Highly intolerant; structural modelling: possibly disrupting side chain construction	ND	ND	SHSY5Y cells: normal, less content		[61]
		c.775A>G p.(Met259Val)	Intolerant	ND	ND	SHSY5Y cells: normal, less content		[61]
		c.776T>C p.(Met259Thr)	Intolerant	ND	ND	SHSY5Y cells: normal, slightly less content		[61]
		c.782T>C p.(Ile261Thr)	Intolerant	ND	ND	SHSY5Y cells: normal, less content		[61]
		c.784G>A p.(Gly262Arg)	Intolerant	ND	ND	SHSY5Y cells: normal, slightly less content		[61]
		c.806G>C p.(Arg269Pro)	Intolerant	ND	ND	SHSY5Y cells: normal, less content		[61]
		c.859G>C p.(Glu287Gln)	Intolerant; structural modelling: closely located to ATP binding site	ND	ND	SHSY5Y cells: normal, less content		[61]
		c.910C>G p.(Arg304Gly)	Intolerant; structural modelling: possibly disrupting polar network building	ND	ND	ND		[61]
		c.910C>T p.(Arg304Trp)	Intolerant; structural modelling: possibly disrupting polar network building	ND	ND	SHSY5Y cells: normal		[61]
		c.915G>T p.(Glu305Asp)	Intolerant	ND	ND	SHSY5Y cells: normal		[61]
		c.929T>C p.(Met310Thr)	Highly intolerant	ND	ND	SHSY5Y cells: normal		[61]
		c.1127+337A>G p.(Ser376Arg15*)	ND	ND	ND	ND	Cryptic exon inclusion introducing a premature termination codon	[60]
		c.1147G>A p.(Glu383Lys)	Neutral; structural modeling: possibly disrupting hydrogen bond network	ND	ND	SHSY5Y cells: normal, slightly less content		[61]
	*PSMC5* (NM_002805.6)	c.166+3del p.?	Structural modelling: located in coiled-coil region	ND	ND	ND		[12]
		c.205C>T p.(Gln69*)	ND	HeLa *PSMC5* siRNA knockdown model: less 26S/30S content (α1-7)	HeLa *PSMC5* siRNA knockdown model: reduction of chymotrypsin-like activity	HeLa and SHYS5Y *PSMC5* siRNA knockdown model: reduced content		[63]
		c.206A>G p.(Gln69Arg)	Tolerated, benign; structural modelling: located in coiled-coil region	ND	ND	ND		[12]
		c.414G>C p.(Met138Ile)	Deleterious, possibly damaging; structural modelling: located in P-loop	ND	ND	ND		[12]
		c.479A>C p.(Gln160Ala)	ND	HeLa *PSMC5* siRNA knockdown model: less 26S content (α-1-7)	HeLa *PSMC5* siRNA knockdown model: reduction of chymotrypsin-like activity	HeLa and SHYS5Y *PSMC5* siRNA knockdown model: reduced content		[63]
		c.548C>T p.(Pro183Leu)	Deleterious, probably damaging; structural modelling: located in AAA-ATPase region	SHSY5Y cells: normal incorporation, less content (HA)	ND	SHSY5Y cells: normal, less content		[12]
		c.587del p.(Lys196Argfs*29)	Structural modelling: located in AAA-ATPase region	ND	ND	ND		[12]
		c.601C>T p.(Arg201Trp)	Deleterious, probably damaging; structural modelling: located in AAA-ATPase region	SHSY5Y cells: normal incorporation (HA)	ND	SHSY5Y cells: normal, less content		[12]
		c.605C>T p.(Ala202Val)	Deleterious, probably damaging; structural modelling: located in AAA-ATPase region	SHSY5Y cells: absent incorporation (HA)	ND	SHSY5Y cells: no expression		[12]
		c.620C>T p.(Thr207Met)	Deleterious, probably damaging; structural modelling: located in AAA-ATPase region	SHSY5Y cells: normal incorporation, increased content (HA)	ND	SHSY5Y cells: normal		[12]
		c.647G>A p.(Gly216Asp)	Deleterious, probably damaging; structural modelling: located in AAA-ATPase region	SHSY5Y cells: normal incorporation, less content (HA)	ND	SHSY5Y cells: normal, less content		[12]
		c.653A>T p.(Glu218Val)	Deleterious, probably damaging; structural modelling: located in AAA-ATPase region	ND	ND	ND		[12]
		c.662A>G p.(Gln221Arg)	Deleterious, probably damaging; structural modelling: located in AAA-ATPase region	SHSY5Y cells: normal incorporation (HA)	ND	SHSY5Y cells: normal		[12]
		c.749A>G p.(Glu250Gly)	Deleterious, probably damaging; structural modelling: located in AAA-ATPase region	ND	ND	ND		[12]
		c.749A>T p.(Glu250Val)	Deleterious, probably damaging; structural modelling: located in AAA-ATPase region	SHSY5Y cells: normal incorporation, less content (HA)	ND	SHSY5Y cells: normal, very less content		[12]
		c.754G>A p.(Asp252Asn)	Deleterious, probably damaging; structural modelling: located in AAA-ATPase region	ND	ND	ND		[12]
		c.772C>T p.(Arg258Trp)	Deleterious, probably damaging; structural modelling: located in AAA-ATPase region	SHSY5Y cells: normal incorporation (HA)	ND	SHSY5Y cells: normal, slightly less content		[12]
		c.773G>A p.(Arg258Gln)	Deleterious, probably damaging; structural modelling: located in AAA-ATPase region	ND	ND	ND		[12]
		c.845G>A p.(Gly282Asp)	Deleterious, probably damaging; structural modelling: located in AAA-ATPase region	ND	ND	ND		[12]
		c.959C>A p.(Pro320His)	Deleterious, probably damaging; structural modelling: located in AAA-ATPase region	SHSY5Y cells: normal incorporation, less content (HA)	ND	SHSY5Y cells: normal, slightly less content		[12]
		c.959C>G p.(Pro320Arg)	Deleterious, probably damaging; structural modelling: located in AAA-ATPase region	HeLa *PSMC5* siRNA knockdown model: less 26S content (α1-7); BE(2)-M17 cells: less content 26S/30S (α1-7); SHSY5Y cells: normal incorporation, very less content (HA)	HeLa *PSMC5* siRNA knockdown model: reduction of chymotrypsin-like activity; BE(2)-M17 cells: higher chymotrypsin-like activity	HeLa and SHYS5Y *PSMC5* siRNA knockdown model: reduced content; HeLa *PSMC5* P320R transfected cells: normal maturation; BE(2)-M17 cells: normal; SHSY5Y cells: normal, very less content		[12,63]
		c.968_969+1del p.?	Structural modelling: located in P-loop	ND	ND	ND	Predicted p.(Glu323del)	[12]
		c.970-2A>G p.?	Structural modelling: located in P-loop	ND	ND	ND	Predicted exon 10 skipping resulting in in-frame deletion	[12]
		c.973C>T p.(Arg325Trp)	Deleterious, probably damaging; structural modelling: located in P-loop	HeLa *PSMC5* siRNA knockdown model: less 26S content (α1-7); SHSY5Y cells: normal incorporation, less content, increase of 19S precursor (HA)	HeLa *PSMC5* siRNA knockdown model: reduction of chymotrypsin-like activity	HeLa and SHYS5Y *PSMC5* siRNA knockdown model: reduced content; SHSY5Y cells: normal, less content		[12,63]
		c.1103T>C p.(Met368Thr)	Deleterious, probably damaging; structural modelling: located in AAA lid domain	SHSY5Y cells: no 26S incorporation, 19S and precursor accumulation (HA)	ND	SHSY5Y cells: normal, less content		[12]
		c. 1158_1159del p.(Val387Serfs*9)	Structural, modelling: located close to C-terminus	ND	ND	ND		[12]
		c. 1177_1179del p. (Lys393del)	Structural modelling: located close to C-terminus	ND	ND	ND		[12]
		c.1182_1183del p.(Asp394Glufs*2)	Structural modelling: located close to C-terminus	SHSY5Y cells: normal, increased content, increase of 19S precursor (HA)	ND	SHSY5Y cells: normal, higher content		[12]
	*PSMD11* (NM_002815.4)	c.268C>T p.(Arg90*)	ACMG classification; structural modelling: Rpn6 molecular clamp between 19S base, lid and 20S	HEK293T siRNA *PSMD11* knockdown cells: normal, less content (Rpn5)	HEK293T siRNA *PSMD11* knockdown cells: reduced chymotrypsin-like activity	ND		[64]
		c.559C>T p.(Arg187*)				ND		[64]
		c.612dup p.(Lys205*)				ND		[64]
		c.619C>T p.(Gln207*)				ND		[64]
		c.788+2T>C p.?				ND	Predicted exon 7 skipping resulting in frameshift	[64]
		c.788C>T p.(Thr263Ile)				ND		[64]
		c.851_854del p.(Thr284Lysfs*3)				ND		[64]
		c.914C>T p.(Ala305Val)				ND		[64]
		c.1009C>T p.(Arg337*)				ND		[64]
		Complete deletion				ND		[64]
	*PSMD12* (NM_002816.5)	c.148_149del p.(Leu50Glyfs*26)	Classification: pathogenic	ND	ND	SHSY5Y *PSMD12* knockout clones: truncated versions of *PSMD12* from 2 *PSMD12* knockout clones		[59]
		c.316C>T p.(Gln106*)	Classification: pathogenic	ND	ND			[59]
		c.333T>G, p.(Tyr111*)	Classification: pathogenic	ND	ND	ND		[65]
		c.367C>T p.(Arg123*)	ND	ND	ND	HeLa cell model: absent expression		[56,57]
		c.435_438del p.(Thr146Lysfs*3)	Classification: pathogenic	ND	ND	SHSY5Y *PSMD12* knockout clones: truncated versions of *PSMD12* from 2 *PSMD12* knockout clones		[59]
		c.446T>G p.(Leu149*)	Classification: pathogenic	ND	ND			[59]
		c.508_509del p.(Gln170Glyfs*40)	Classification: pathogenic	ND	ND			[59]
		c.526del p.(Ser176Glnfs*15)	Classification: pathogenic	ND	ND			[59]
		c.544C>T p.(Arg182*)	Classification: pathogenic	ND	ND			[59]
		c.601C>T p.(Arg201*)	ND	ND	ND	ND		[56], [57]
		c.795+1G>A p.?	Classification: pathogenic	ND	ND	SHSY5Y *PSMD12* knockout clones: truncated versions of *PSMD12* from 2 *PSMD12* knockout clones		[59]
		c.865C>T p.(Arg289*)	Classification: pathogenic and damaging	HEK293T *PSMD12* knockdown cells: normal 26S incorporation, but less content; absent 30S incorporation (β7)	HEK293T cells: reduction of all activities	HEK293T cells: only truncated protein (FLAG)		[58,65]
		c.906C>A p.(Tyr302*)	Classification: pathogenic	ND	ND	SHSY5Y *PSMD12* knockout model: truncated versions of *PSMD12* from 2 *PSMD12* knockout clones		[59]
		c.909-2A>G p.?	ND	ND	ND	ND		[56]
		c.937G>T p.(Glu313*)	Classification: pathogenic	ND	ND	SHSY5Y *PSMD12* knockout model: truncated versions of *PSMD12* from 2 *PSMD12* knockout clones		[59]
		c.1033G>T p.(Glu345*)	Classification: pathogenic	ND	ND			[59]
		c.1060_1061del p.(Leu354Glufs*6)	Classification: pathogenic	ND	ND			[59]
		c.1071_1072del p.(Arg357Serfs*3)	Classification: pathogenic	ND	ND			[59]
		c.1083+1G>A p.?	Classification: pathogenic	ND	ND			[59]
		c.1162-1G>A p.?	Classification: pathogenic	ND	ND			[59]
		c.1246C>T p.(Gln416*)	Classification: pathogenic	ND	ND			[59]
		c.1274T>G p.(Leu425*)	Classification: pathogenic	ND	ND	HeLa cell model: slight shortened protein, presence of HMW protein		[56]
		c.1300del p.(Ser434Hisfs*2)	Classification: pathogenic	ND	ND	SHSY5Y *PSMD12* knockout model: truncated versions of *PSMD12* from 2 *PSMD12* knockout clones		[59]
		Partial and complete deletions	Classification: pathogenic	ND	ND			[[56], [59]]

Associated with ID or DD								

	Gene (transcript)	HGVS	Experimental outcome				Additional information	Ref
			Genetic/structural prediction^a^	Cell model				
				Proteasome biogenesis	Proteasome activity/function	Affected protein maturation		

20S (Standard)	*PSMA7* (NM_002792.4)	c.335C>A p.(Ala112Asp)	ND	ND	ND	ND		[68]
19S	*PSMC1* (NM_002802.3)	Complete deletion	ND	ND	ND	ND		[66]

ACMG, American College of Medical Genetics and Genomics; DD, developmental delay (based on reference); EMBL-EBI, European Bioinformatics Institute; HGVS, Human Genome Variation Society; ID, intellectual disability (based on reference); HMW, high molecular weight; LCL, lymphoblastoid cell line; NCBI, National Center for Biotechnology Information; ND, not defined; PRAAS, proteasome-associated autoinflammatory syndrome; PRNDD, proteasome-associated neurodevelopmental disorder; proteasome biogenesis relates to native polyacrylamide gel electrophoresis (PAGE) analysis of proteasome complexes and glycerol gradient experiments; PROVEAN, Protein Variation Effect Analyzer; Ref, reference; Rpt, regulatory particle ATPase; SCID, severe combined immunodeficiency; SIFT, Scale-Invariant Feature Transform.

Variants were annotated in accordance with HGVS 3′-aligned (right-normalized) nomenclature, using the appropriate MANE (Matched Annotation from NCBI and EMBL-EBI) transcripts to ensure consistent and standardized variant representation.

V5, myc, HA, and FLAG have been used as tags to stain for proteasome variants.

a Refers to bioinformatic tools such as Polyphen2, PROVEAN, and SIFT; classification based on ACMG regulations.

This in turn complicates the diagnostic profiling of these patients. A sufficient diagnosis encompasses the clinical manifestations, genetic identification of the variant(s) and its structural and functional consequences at the molecular level.

### Genetic background of proteasomopathies

Most current patients with PRAAS carry homozygous or (compound) heterozygous recessively inherited variants, frequently detected in the 20S CP of the proteasome (Table 2).

The Hispanic founder mutation *PSMB8* p.T75M and other variants in *PSMB8* represent the most well-known genetic association with PRAAS [[25], [26], [27], [28], [29], [30],^32^,^34^,^39^,^40^,^42^,[45], [46], [47], [48], [49],[51], [52], [53]]. Additional genes within the 20S CP have been identified, including *PSMA3, PSMA5, PSMA6, PSMB2, PSMB4, PSMB9, PSMB10*, and *PSME2* with a recessive inheritance pattern, surprisingly often resulting in an additive LOF (Table 3) [30,36,39,43,50,52]. For the inherited variants of *PSMB2* and *PSME2* [50], clear evidence of its pathogenicity has not been proven yet, and additional studies are required to fully understand their impact. Nevertheless, novel heterozygous variants in all immunoproteasome-specific subunits have been considered dominant-negative [11,31,35,44,50,51,54]. So far, variants in only 2 genes encoding 20S proteasome assembly helpers, in particular *PSMG2* and *POMP*, were associated with PRAAS. Whereas the described *PSMG2* variant showed a recessive inheritance, all described lesions in *POMP* occurred de novo and act in a dominant-negative fashion [30,33,37,41]. Up to now, only 1 PRAAS case carried a variant within the 19S RP—*PSMC5* together with 2 other 20S related mutations (*PSMA5* and *PSMB8*) [39].

**Table 3:** List of published PRAAS, PRNDD, and ID/DD cases carrying variants in proteasome subunits and related assembly helpers.

PRAAS and SCID						
Gene	HGVS	Inheritance	Experimental outcome			Ref
			Proteasome biogenesis	Proteasome activity/function	Affected protein maturation	
*PSMA3 PSMB8*	c.404+2T>C p.(His111Phefs*10), c.224C>T p.(Thr75Met)	Heterozygous	ND	ND	ND	[30]
*PSMA3*^b^*PSMB8*	c.696_698del^b^ p.(Arg233del), c.224C>T p.(Thr75Met)	Heterozygous, bde novo	Fibroblasts: reduced content (α6); normal incorporation, reduced content (β5i); EBV-B cells: normal incorporation, reduced 26S content (β5i); Keratinocytes: reduced content (α6); normal incorporation, reduced content (β5i)	Reduction of all activities in PBMCs, keratinocytes and EBV-B cells	EBV-B cells: normal for both, but slightly less content for α7	[30]
*PSMA5*^b^*PSMB8 PSMC5*	c.502C>T p.(Arg168*)^b^, c.224C>T p.(Thr75Met), c.1080+1_1080+10del p.(Ala324_Lys360)	Heterozygous, bde novo	ND	ND	ND	[39]
*PSMA6 PSMB8*	c.171+64_253+528del, c.224C>T p.(Thr75Met)	Heterozygous	ND	PBMCs: reduction of all activities	ND	[52]
*PSMB2 PSMB10*^b^*PSME2*	c.142G>A p.(Gly48Arg), c.601G>A p.(Gly201Arg)^b^, c.512G>A p.(Arg171His)	Heterozygous, bde novo	ND	ND	PSMB2: normal (PBMCs, EBV-B cells, fibroblasts), but reduced content in EBV-B cells PSME2: normal, but reduced content (PBMCs, EBV-B cells, fibroblasts) PSMB10: normal, but reduced content (PBMCs, EBV-B cells, fibroblasts); higher precursor content (EBV-B cells)	[50]
*PSMB4*	c.231del p.(Leu78Trpfs*31), c.494+17A>G p.?	Compound heterozygous	T cells: less 26S/30S content and higher precursor content (α6)	T cells: reduction of chymotrypsin-like activity	T cells: normal, but less content	[36]
*PSMB4*	c.-9G>A p.?, c.636_644del p.(Asp212_Val214del)	Compound heterozygous	Fibroblasts and EBV-B cells: higher precursor and 30S content (α6) Keratinocytes: less 20S content, slightly higher 30S content (α6 and β7)	PBMCs, EBV-B cells and keratinocytes: reduction of all activities	EBV-B cells: normal	[30]
*PSMB4 PSMB8*	c.666C>A p.(Tyr222*), c.313A>C p.(Lys105Gln)	Heterozygous	ND	ND	ND	[30]
*PSMB4 PSMB9*	c.44dup p.(Pro16Serfs*45), c.494G>A p.(Gly165Asp)	Heterozygous	Keratinocytes: normal 26S/30S incorporation, less 26S content and absent 20S content (α6); normal incorporation, but less 20S/26S content (β1i)	PBMCs: reduced caspase-like and tryptic activity; keratinocytes: reduction of all activities	ND	[30]
*PSMB8*	c.163C>T p.(Gln55*), c.352T>C p.(Ser118Pro)	Compound heterozygous	ND	ND	ND	[39,48]
*PSMB8*	c.224C>T p.(Thr75Met)	Homozygous	Keratinocytes: normal incorporation (α6, β5i); EBV-B cells: normal incorporation (α6, β5i), slightly reduced 30S content (β5i)	Lymphoblasts: reduced trypsin- and chymotrypsin-like activity, slight increase in caspase-like activity; PBMCs: reduced chymotrypsin-like activity; increase of caspase- and trypsin-like activity; keratinocytes: reduced chymotrypsin-like and caspase-like activity; EBV-B cells: reduction of chymotrypsin-like activity, increase of trypsin-like activity	Lymphoblasts: normal; EBV-B cells: normal, slight presence of proform	[25,30,40,53]
*PSMB8*	c.224C>T p.(Thr75Met)	Heterozygous	ND	ND	ND	[40]
*PSMB8*	c.224C>T p.(Thr75Met), c.274G>A p.(Ala92Thr)	Heterozygous	ND	PBMCs: increase caspase- and tryptic-like activity, reduced chymotrypsin-like activity	ND	[26,30]
*PSMB8*	c.269C>T p.(Ser90Phe)	Heterozygous, de novo	ND	ND	ND	[51]
*PSMB8*	c.274G>A p.(Ala92Thr)	Homozygous	ND	ND	ND	[28,45]
*PSMB8*	c.275C>T p.(Ala92Val), c.313A>C p.(Lys105Gln)	Compound heterozygous	ND	ND	ND	[34]
*PSMB8*	c.280G>C p.(Ala94Pro)	Homozygous	ND	ND	ND	[32]
*PSMB8*	c.349A>G p.(Met117Val)	Homozygous	ND	ND	ND	[46]
*PSMB8*	c.367G>A p.(Asp123Asn), c.385C>T p.(Arg129Cys)	Compound heterozygous	ND	ND	ND	[27]
*PSMB8*	c.389del p.(Leu130Argfs*29), c.602G>T p.(Gly201Val)	Compound heterozygous	ND	Monocytes: reduced chymotrypsin-like activity	ND	[47]
*PSMB8*	c.405C>A p.(Cys135*)	Homozygous	Fibroblasts: presence of precursors (α6); no incorporation (β5i)	PBMCs: reduction of all activities	ND	[30,40]
*PSMB8*	c.602G>T p.(Gly201Val)	Homozygous	EBV-B cells: higher precursor content (α6); LCLs: presence of precursors (α6); normal incorporation, presence of immature form (β5i)	EBV-B cells: reduced chymotrypsin-like activity; LCLs: reduction of all activities	EBV-B cells: normal, but reduced content	[29,42]
*PSMB8*	c.625G>A, p.(Gly209Arg)	Heterozygous, de novo	T cells: normal in β5i, but less content for 20S/26S and accumulation of precursors (α6)	T cells: slightly reduced for chymotrypsin-like activity, reduction of pan-activity	T cells: normal	[11]
*PSMB8*	c.625G>C, p.(Gly209Arg)	Heterozygous, de novo	T cells: normal in β5i, but less content for 20S/26S and accumulation of precursors (α6)	T cells: slightly reduced for chymotrypsin-like activity, reduction of pan-activity	T cells: normal	[11]
*PSMB8*	c.704C>A p.(Ala235Asp)	Heterozygous, de novo	Fibroblasts: possibly assembly defect (Mass Spectrometry)	ND	ND	[51]
*PSMB9*	c.467G>A p.(Gly156Asp)	Heterozygous, de novo	EBV-B cells: normal incorporation, but less overall content and accumulation in intermediate fractions (α6, β1i)	EBV-B cells: reduction of chymotrypsin-like activity; LCLs: reduction of all activities	EBV-B cells: normal, but less content and presence of immature form; LCLs: absent maturation, presence of immature forms	[31,44]
*PSMB10*	c.40_42del p.(Phe14del), c.247dup p.(Cys83Leufs*123)	Compound heterozygous	ND	ND	ND	[39]
*PSMB10*	c.40_42del p.(Phe14del), c.500G>A p.(Gly167Asp)	Compound heterozygous	ND	ND	ND	[39]
*PSMB10*	c.40_42del p.(Phe14del), c.710+1G>C p.?	Compound heterozygous	ND	ND	ND	[39]
*PSMB10*	c.41T>C p.(Phe14Ser)	Homozygous	ND	PBMCs: reduction of all activities	ND	[43]
*PSMB10*	c.166G>C p.(Asp56His)	Heterozygous, de novo	ND	ND	Fibroblasts: normal, but presence of intermediate form upon IFN-γ treatment	[35]
*PSMB10*	c.601G>A p.(Gly201Arg)	Heterozygous, de novo	ND	ND	Fibroblasts: normal, but presence of intermediate form upon IFN-γ treatment	[35]
*PSMB10*	c.601G>C p.(Gly201Arg)	Heterozygous, de novo	ND	ND	Fibroblasts: normal, but presence of intermediate form upon IFN-γ treatment	[35]
*PSMB10*	c.614A>C p.(Asp205Ala)	Heterozygous, de novo	ND	ND	ND	[54]
*PSMB10*	c.623C>T p.(Ser208Phe)	Heterozygous, de novo	T cells: higher proteasome content (α6), less proteasome content in complex with PA28α	T cells: reduced chymotrypsin-like activity	T cells: normal, but slightly less content	[54]
*POMP*	c.326dup p.(Asp109Glufs*2)	Heterozygous, de novo	ND	Fibroblasts: reduced chymotrypsin-like activity	Fibroblasts: normal, but less content and presence of truncated version; PBMCs: normal, but presence of truncated version (pre-HSCT)	[33]
*POMP*	c.334_335del p.(Ile112Trpfs*3)	Heterozygous, de novo	Fibroblasts: overall less proteasome content, increase of precursors (α6)	PBMCs: minor reduction of tryptic-like activity	PBMCs: normal, less content and presence of truncated version; Fibroblasts: normal, less content and presence of truncated version	[37,38]
*POMP*	c.340_344dup p.(Glu115Aspfs*20)	Heterozygous, de novo	ND	ND	ND	[30]
*POMP*	c.342_348delinsACC p.(Phe114Leufs*18)	Heterozygous, de novo	Fibroblasts: overall less proteasome content, slight increase of precursors (α6)	PBMCs: reduced caspase- and chymotrypsin-like activity	PBMCs and EBV-B cells: normal, but increase in content and presence of truncated version; Fibroblasts: normal, less content and presence of truncated version	[37,38]
*PSMG2*	c.666_667del p.(Tyr223Serfs*2), c.675T>G p.(Asn225Lys)	Compound heterozygous	Fibroblasts: reduced incorporation, less proteasome content (PAC1/2, α6)	Fibroblasts: reduction of all activities	Fibroblasts: normal, but less content	[41]

PRNDD						
Gene	HGVS	Inheritance	Experimental outcome			Ref
			Proteasome biogenesis	Proteasome activity/function	Affected protein maturation	
*PSMB1*	c.307T>C p.(Tyr103His)	Homozygous	ND	ND	ND	[67]
*PSMC1*	c.983T>C p.(Ile328Thr)	Homozygous	ND	ND	ND	[62]
*PSMC3*	c.511C>T p.(Arg171Trp)	Heterozygous, de novo	ND	ND	ND	[61]
*PSMC3*	c.523A>G p.(Met175Val)	Heterozygous, de novo	ND	ND	ND	[61]
*PSMC3*	c.686C>T p.(Pro229Leu)	Heterozygous, de novo	ND	ND	ND	[61]
*PSMC3*	c.710C>T p.(Ala237Val)	Heterozygous, de novo	ND	ND	ND	[61]
*PSMC3*	c.775A>G p.(Met259Val)	Heterozygous, de novo	ND	ND	ND	[61]
*PSMC3*	c.776T>C p.(Met259Thr)	Heterozygous, de novo	ND	ND	ND	[61]
*PSMC3*	c.782T>C p.(Ile261Thr)	Heterozygous, de novo	ND	ND	ND	[61]
*PSMC3*	c.784G>A p.(Gly262Arg)	Heterozygous, de novo	T cells: normal incorporation (Rpt5)	T cells: normal chymotrypsin-like activity	T cells: normal	[61]
*PSMC3*	c.806G>C p.(Arg269Pro)	Heterozygous, de novo	ND	ND	ND	[61]
*PSMC3*	c.859G>C p.(Glu287Gln)	Heterozygous, de novo	ND	ND	ND	[61]
*PSMC3*	c.910C>G p.(Arg304Gly)	Heterozygous, de novo	ND	ND	ND	[61]
*PSMC3*	c.910C>T p.(Arg304Trp)	Heterozygous, de novo	T cells: normal incorporation, very less content for 26S (α6, Rpt5)	T cells: reduced chymotrypsin-like activity	T cells: normal	[61]
*PSMC3*	c.915G>T p.(Glu305Asp)	Heterozygous, de novo	T cells: less content for 26S (Rpt5)	T cells: reduced chymotrypsin-like activity	T cells: normal	[61]
*PSMC3*	c.929T>C p.(Met310Thr)	Heterozygous, de novo	ND	ND	ND	[61]
*PSMC3*	c.1127+337A>G p.(Ser376Arg15*)	Homozygous	Fibroblasts: increased incorporation (Rpt5)	Fibroblasts: slight increase of chymotrypsin-like activity	Fibroblasts: normal, presence of truncated protein	[60]
*PSMC3*	c.1147G>A p.(Glu383Lys)	Heterozygous, de novo	ND	ND	ND	[61]
*PSMC5*	c.166+3del p.?	Heterozygous, de novo	ND	ND	ND	[12]
*PSMC5*	c.205C>T p.(Gln69*)	Heterozygous, de novo	ND	ND	ND	[63]
*PSMC5*	c.206A>G p.(Gln69Arg)	Heterozygous, de novo	ND	ND	ND	[12]
*PSMC5*	c.414G>C p.(Met138Ile)	Heterozygous, de novo	ND	ND	ND	[12]
*PSMC5*	c.479A>C p.(Gln160Ala)	Heterozygous	ND	ND	ND	[63]
*PSMC5*	c.548C>T p.(Pro183Leu)	Assumed de novo	ND	ND	ND	[12]
*PSMC5*	c.587del p.(Lys196Argfs*29)	Heterozygous, inherited from affected mother	ND	ND	ND	[12]
*PSMC5*	c.601C>T p.(Arg201Trp)	Assumed de novo	T cells: less 26S content (Rpt6)	T cells: normal chymotrypsin-like activity	T cells: normal, presence of LMM form	[12]
*PSMC5*	c.605C>T p.(Ala202Val)	Heterozygous, de novo	ND	ND	ND	[12]
*PSMC5*	c.620C>T p.(Thr207Met)	Homozygous	ND	ND	ND	[12]
*PSMC5*	c.647G>A p.(Gly216Asp)	Heterozygous, de novo	ND	ND	ND	[12]
*PSMC5*	c.653A>T p.(Glu218Val)	Heterozygous, de novo	ND	ND	ND	[12]
*PSMC5*	c.749A>G p.(Glu250Gly)	Heterozygous, de novo	ND	ND	ND	[12]
*PSMC5*	c.749A>T p.(Glu250Val)	Heterozygous, de novo	T cells: normal incorporation (α6, Rpt6)	T cells: normal chymotrypsin-like activity	T cells: normal, presence of slight shorter form	[12]
*PSMC5*	c.754G>A p.(Asp252Asn)	Heterozygous, de novo	ND	ND	ND	[12]
*PSMC5*	c.772C>T p.(Arg258Trp)	Heterozygous, de novo	ND	ND	ND	[12]
*PSMC5*	c.773G>A p.(Arg258Gln)	Heterozygous, inherited from affected mother	ND	ND	ND	[12]
*PSMC5*	c.845G>A p.(Gly282Asp)	Heterozygous, de novo	ND	ND	ND	[12]
*PSMC5*	c.959C>A p.(Pro320His)	Assumed de novo	ND	ND	ND	[12]
*PSMC5*	c.959C>G p.(Pro320Arg)	Heterozygous, de novo	T cells: less 26S content (α6, Rpt6)	T cells: slightly reduced chymotrypsin-like activity	T cells: normal, less content and presence of LMM form	[12,63]
*PSMC5*	c.968_969+1del p.?	Heterozygous, de novo	ND	ND	ND	[12]
*PSMC5*	c.970-2A>G p.?	Assumed de novo	ND	ND	ND	[12]
*PSMC5*	c.973C>T p.(Arg325Trp)	Heterozygous, de novo	T cells: less 26S content (α6, Rpt6)	T cells: reduced chymotrypsin-like activity	T cells: normal, presence of LMM form	[12,63]
*PSMC5*	c.1103T>C p.(Met368Thr)	Heterozygous, de novo	ND	ND	ND	[12]
*PSMC5*	c. 1158_1159del p.(Val387Serfs*9)	Heterozygous, de novo	ND	ND	ND	[12]
*PSMC5*	c. 1177_1179del p. (Lys393del)	Heterozygous, de novo	ND	ND	ND	[12]
*PSMC5*	c.1182_1183del p.(Asp394Glufs*2)	Heterozygous, de novo	ND	ND	ND	[12]
*PSMD11*	c.268C>T p.(Arg90*)	Heterozygous, inherited from affected father	ND	ND	ND	[64]
*PSMD11 PSMC5*^b^	c.268C>T p.(Arg90*), c.662A>G p.(Gln221Arg)^b^	Heterozygous, inherited from affected father, bde novo	T cells: less 26S content (α6, Rpt6)	T cells: normal chymotrypsin-like activity	T cells: normal, presence of LMM form	[12]
*PSMD11*	c.559C>T p.(Arg187*)	Heterozygous, de novo	ND	ND	ND	[64]
*PSMD11*	c.612dup p.(Lys205*)	Heterozygous, de novo	T cells: normal 26S content (α6)	T cells: normal chymotrypsin-like activity	T cells: normal, less content, presence of phosphorylated form	[64]
*PSMD11*	c.619C>T p.(Gln207*)	Heterozygous, de novo	ND	ND	ND	[64]
*PSMD11*	c.788+2T>C p.?	Heterozygous, inherited from mother	T cells: less content for 26S (α6)	T cells: normal chymotrypsin-like activity	T cells: normal, less content, presence of phosphorylated form	[64]
*PSMD11*	c.788C>T p.(Thr263Ile), c.914C>T p.(Ala305Val)	Compound heterozygous	T cells: less content for 26S (α6)	T cells: slightly reduced chymotrypsin-like activity	T cells: normal, increase of content, presence of phosphorylated form	[64]
*PSMD11*	c.851_854del p.(Thr284Lysfs*3)	Heterozygous, de novo	ND	ND	ND	[64]
*PSMD11*	c.1009C>T p.(Arg337*)	Heterozygous, de novo	ND	ND	ND	[64]
*PSMD11*	complete deletion	Heterozygous, de novo	ND	ND	ND	[64]
*PSMD12*	c.148_149del p.(Leu50Glyfs*26)	Heterozygous, de novo	ND	ND	ND	[59]
*PSMD12*	c.316C>T p.(Gln106*)	Heterozygous, de novo	ND	ND	ND	[59]
*PSMD12*	c.333T>G p.(Tyr111*)	Heterozygous, de novo	ND	ND	ND	[65]
*PSMD12*	c.367C>T p.(Arg123*)	Heterozygous, de novo	ND	PBMCs: normal chymotrypsin-like activity	PBMCs: normal, very less content	[56,57]
*PSMD12*	c.435_438del p.(Thr146Lysfs*3)	Heterozygous, de novo	ND	ND	ND	[59]
*PSMD12*	c.446T>G p.(Leu149*)	Heterozygous, de novo	ND	ND	ND	[59]
*PSMD12*	c.508_509del p.(Gln170Glyfs*40)	Heterozygous, de novo	ND	ND	ND	[59]
*PSMD12*	c.526del p.(Ser176Glnfs*15)	Heterozygous, de novo	ND	ND	ND	[59]
*PSMD12*	c.544C>T p.(Arg182*)	Heterozygous, de novo	ND	ND	ND	[59]
*PSMD12*	c.601C>T p.(Arg201*)	Heterozygous, de novo	ND	ND	ND	[56,57]
*PSMD12*	c.795+1G>A p.?	Heterozygous, de novo	ND	ND	ND	[59]
*PSMD12*	c.865C>T p.(Arg289*)	Heterozygous, de novo	PBMCs: less 26S content (α4, Rpn5)	PBMCs: reduction of all activities	PBMCs: normal, less content	[58,65]
*PSMD12*	c.906C>A p.(Tyr302*)	Heterozygous, de novo	ND	ND	ND	[59]
*PSMD12*	c.909-2A>G p.?	Heterozygous, de novo	ND	ND	ND	[56]
*PSMD12*	c.937G>T p.(Glu313*)	Heterozygous, de novo	ND	ND	ND	[59]
*PSMD12*	c.1033G>T p.(Glu345*)	Heterozygous, de novo	ND	ND	ND	[59]
*PSMD12*	c.1060_1061del p.(Leu354Glufs*6)	Heterozygous, de novo	ND	ND	ND	[59]
*PSMD12*	c.1071_1072del p.(Arg357Serfs*3)	Heterozygous	ND	ND	ND	[59]
*PSMD12*	c.1083+1G>A p.?	Heterozygous, de novo	ND	ND	ND	[59]
*PSMD12*	c.1162-1G>A p.?	Heterozygous, de novo	ND	ND	ND	[59]
*PSMD12*	c.1246C>T p.(Gln416*)	Heterozygous, de novo	ND	ND	ND	[59]
*PSMD12*	c.1274T>G p.(Leu425*)	Heterozygous, de novo	ND	ND	ND	[56]
*PSMD12*	c.1300del p.(Ser434Hisfs*2)	Heterozygous, de novo	ND	ND	ND	[59]
*PSMD12*	Partial and complete deletions	Heterozygous	BLCLs: normal incorporation, less content 26S/30S (Rpn5)	BLCLs: reduced chymotrypsin-like activity	BLCLs: normal, very less content	[56], [59]

Associated with ID or DD						

Gene	HGVS	Inheritance	Experimental outcome			Ref
			Proteasome biogenesis	Proteasome activity/function	Affected protein maturation	

PSMA7	c.335C>A p.(Ala112Asp)	de novo	ND	ND	ND	[68]
*PSMC1*	Complete deletion	Heterozygous	ND	ND	ND	[66]

ACMG, American College of Medical Genetics and Genomics; BLCLs, B-Lymphoblastoid Cell Line; DD, developmental delay (based on reference); EBV-B cells, Epstein-Barr Virus transformed B cells; EMBL-EBI, European Bioinformatics Institute; HGVS, Human Genome Variation Society; ID, intellectual disability (based on reference); IFN, interferon; LCL, lymphoblastoid cell line; NCBI, National Center for Biotechnology Information; LMM, low molecular weight; ND, not defined; PBMCs, peripheral blood mononuclear cells; PRAAS, proteasome-associated autoinflammatory syndrome; PRNDD, proteasome-associated neurodevelopmental disorder; PROVEAN, Protein Variation Effect Analyzer; proteasome biogenesis relates to native PAGE analysis of proteasome complexes; Ref, reference; SIFT, Scale-Invariant Feature Transform.

Variants were annotated in accordance with HGVS 3′-aligned (right-normalized) nomenclature, using the appropriate MANE (Matched Annotation from NCBI and EMBL-EBI) transcripts to ensure consistent and standardized variant representation.

b de novo*.*

In contrast, PRNDD-associated subunit variants are primarily located within the 19S RP genes and are often de novo heterozygous. Variants in *PSMC1, PSMC3, PSMC5, PSMD11*, and *PSMD12*, typically exhibit a dominant-negative effect and have been reported de novo with only minor exceptions (Table 3) [12,[56], [57], [58], [59], [60], [61], [62], [63], [64], [65]]. Two cases describing a de novo heterozygous variant in the 20S CP gene *PSMA7* and a homozygous *PSMB1* variant break the rules of this rationale and suggest that the phenotypic manifestation may occur independent of the subunit localisation in certain subcomplexes [67,68].

### Structure-function analysis in proteasomopathies

Nowadays, genetic tools already provide insights into functional consequences or mRNA stability; nevertheless, the improvement of genetic tools often fails to predict pathogenicity resulting in variants of uncertain significance (VUS).

In the context of the sophisticated proteasome system in mammals with a modular architecture of alternative subunits and regulators, the interplay between the structural aspects of proteasome biogenesis and the genetic background of proteasome variants enhances our understanding of the molecular impacts of these variants. More recently, the molecular mechanisms including conformational changes necessary for a fully matured and active proteasome including some of the isoforms have been unravelled by experimentally obtained structures of different proteasome isoforms and assembly intermediates [76,83,84,91,92,[77], [78], [79], [80], [81], [82],[85], [86], [87], [88], [89], [90]] (Table 1). These structures can now be used for structural modelling as a better prediction tool for the pathogenicity of VUS in proteasomopathies.

A clustering of variants in immunosubunits has been observed for PRAAS preferentially impacting the haematopoietic system and partially explaining systemic inflammation [162]. Nevertheless, PRAAS is not restricted to immunoproteasome-specific variants [30,36,39,55].

Beyond the specific roles and distributions of proteasome isoforms in various tissues, with 41 variants located in the 20S CP and 6 in the assembly helpers (Table 2) are primarily associated with recessive inheritance to PRAAS [[25], [26], [27], [28], [29], [30],^32^,^34^,^36^,[39], [40], [41], [42], [43],[45], [46], [47], [48], [49],^52^,^53^]. The minority of these PRAAS-related variants exhibits a dominant-negative behaviour [11,31,33,35,37,44,50,51,54]. The additive effect of 2 or 3 variants, typically resulting in LOF, drives the phenotypic manifestations [30,39]. The well-known *PSMB8* variant, p.Thr75Met, causing a milder PRAAS phenotype, is located in close proximity to the active site Thr73, and acts intramolecularly in the structure mainly impacting only *PSMB8*/β5i [25]. Structural and experimental analysis of this threonine-to-methionine substitution indicates impaired processing and assembly, particularly due to the nonpolar nature of both amino acid residues [30]. However, with 2 heterozygous variants, only 6.25% wild-type proteasomes can be formed and explain healthy carriers with only one variant [30]. By contrast, severe molecular characteristics are often associated with assembly or maturation defects in proteasome biogenesis [76,83]. They highlighted the intricacy of intersubunit interface connections and interactions, suggesting that their disruption can lead to severe assembly defects [76,83]. This is often accompanied by reduced protein expression or impaired maturation of the subunit itself (Table 2). Interestingly, even a heterozygous or compound heterozygous PRAAS-related variant could cause detrimental implications on proteasome biogenesis as shown for *PSMA3, PSMA5, PSMB4* and *PSMB10* [30,39].

Noticeably, the majority of published dominant-negative variants in the 20S CP, such as for *PSMB8, PSMB9*, and *PSMB10*, are commonly located near structural interfaces [31,35,44,50,51]. Of note, the role of the inherited *PSMB2* and *PSME2* variants in [50] is still not well understood. Regarding one of the *PSMB8* dominant-negative variants (eg, *PSMB8* p.Gly209Arg) and the *PSMB9* variant, a steric clash between the β-β-interface probably hinders 20S CP biogenesis, even when 1 subunit is affected [11,31,51]. Additionally, a dramatic dominant-negative phenotype is caused by heterozygous variants in *POMP* [30,33,37]. Here, the assembly helper POMP may lose its ability to interact with the β-subunits during assembly, leading to an early assembly stop [37,38].

Of note, the heterozygous *PSMB8* p.Ser90Phe variant is also located close to the active site and propeptide cleavage site [51]. In comparison to the closely located recessive variants, Phe90 possibly affects the function and structure by introducing an enhanced stiffness in this region, driving the dominant-negative behaviour [51].

In contrast, de novo dominant-negative missense variants are largely observed examining the 19S RP of the 26S proteasome in PRNDD-related cases. Considering the spread of the variant locations within the proteasomal genes, mutations in functional domains such as the AAA-ATPase domain in *PSMC3* and *PSMC5*-related cases hint to disturbances in proteasome substrate translocation from the 19S RP to the 20S CP and possible 26S fusion defects [12,61]. *PSMD11* variants exhibited 26S proteasome assembly defects, which is possibly due to its clamp function between the 19S base and lid [64]. For *PSMD12*, truncating variants result in lower protein expression, leading to haploinsufficiency [56,57]. Importantly, the catalytic peptide-hydrolysing activity is not inhibited *per se*; nevertheless, *PSMC3* and *PSMC5*-related variants impair the ubiquitin-dependent substrate degradation capacity by disrupting 26S/30S biogenesis and therefore proteasome function (Table 2, Table 3). In this context, it is important to note that the E3 ligase HERC1 has been shown as a setscrew in the *PSMC5* protein quality control process prior to its incorporation, which additionally contributes to their lower incorporation profiles [163].

So far, we have summarised that single variant consequences within the proteasome complex is promoted by a detailed mechanistic understanding of the proteasome biogenesis. However, a defined conclusion about their outcome is challenged by compensatory mechanisms such as the heterozygous presence of the wild-type allele. Its contribution to a functional and structural rescue reduces the probability of mutant incorporation into proteasomes [30]. Moreover, variants resulting in skewed gene expression or those undergoing nonsense-mediated decay can be replaced by their subunit counterparts when affecting the active site (eg, *PSMB8* p.C135*) [30,40]. Nonetheless, additional compensatory mechanisms or rescue strategies may even occur at an early developmental stage, which in turn requires further studies.

Considering proteasome biogenesis alone in structural analysis cannot accurately predict the clinical manifestations and outcome of proteasomopathies due to their high complexity. The combination of resolved proteasome structures (Table 1) and artificial intelligence-based structural modelling provides a powerful diagnostic tool for estimating single proteasome variants by improving the prediction of assembly or maturation deficiencies based on variant localisation.

## MOLECULAR CONSEQUENCES OF PROTEASOMOPATHIES IN CELLS

Despite the clinical classification in PRAAS and PRNDD, the molecular signatures of cells from proteasomopathy patients of either phenotype are surprisingly similar. Variants within proteasome isoforms or in certain proteasome subcomplexes may be relevant to tissue and cell specificity, or distinct cellular processes. In PRNDD, manifestation of the disease in the central nervous system may be explained by a certain function of 19S particles independent of protein degradation in neuronal synapses [164].

Positive interferon (IFN) signatures in PRAAS and PRNDD have been detected in most cases [12,18,54,61,64,165], indicating a chronic sterile inflammation in both phenotypes. Indeed, the presented autoinflammatory symptoms in patients with PRAAS are responsive to JAK inhibition reflecting an IFN-driven disease. Despite a positive *in vitro* IFN signature, patients with PRNDD do not exhibit systemic autoinflammatory signs. Type I IFN signalling has been shown to impact stemness, developmental or differentiation processes [[166], [167], [168], [169]]; however, the contribution of IFN in PRNDD is not fully understood.

**Figure 4:**
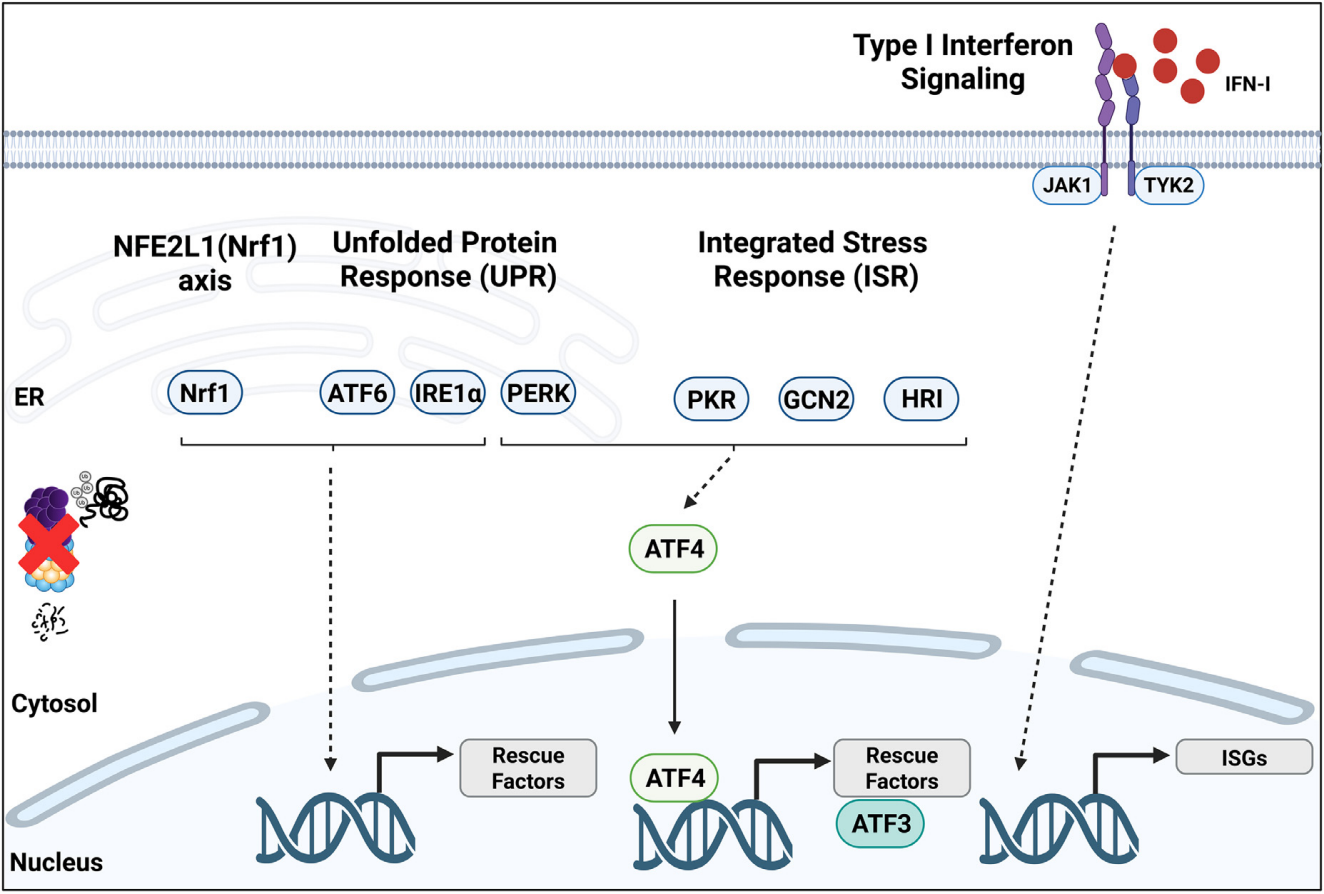
Activated type I interferon (IFN) signalling and stress pathways upon proteasome dysfunction. The NFE2L1 (Nrf1) axis, the unfolded protein response (UPR), and integrated stress response (ISR) as well as the type I IFN signalling has been shown to be activated due to proteasome dysfunction. Nrf1, ATF6, and IRE1α initiate the transcription of rescue factors. ISR and PERK promote ATF4 transcription, which results in the transcription of rescue factors and ATF3. Type I IFNs activate a cascade to induce interferon-stimulated genes (ISGs) expression. Created in BioRender. Venz, S. (2025) https://BioRender.com/p0exv6b. NFE2L1, nuclear factor erythroid-2-like 1; Nrf1, nuclear factor erythroid 2-related factor 1; ATF6, activating transcription factor 6; PERK, protein kinase R (PKR)-like endoplasmic reticulum (ER) kinase; ATF4, activating transcription factor 4, ATF3, activating transcription factor 3; IRE1α, inositol-requiring enzyme 1 alpha.

As expected, proteasome dysfunction leads to imbalanced protein homeostasis and thus affects multiple cellular processes. Impairment of proteasome function has been demonstrated in both PRAAS and PRNDD, resulting in protein aggregate formation. Such proteostatic imbalances are closely connected to the activation of feedback regulation through at least 2 major proteotoxic stress pathways—the unfolded protein response (UPR) and the integrated stress response (ISR). The perturbed protein degradation results in activation of several kinases through distinct molecular triggers. The UPR and ISR kinases IRE1α, PERK, ATF6, PKR, GCN2, HRI, or the transcription factors NFE2L1 and ATF4 initiate the activation of rescue factors (Fig 4) [20,170,171]. In addition, siRNA-mediated knockdown of *PSMB4* in mouse brown preadipocytes as well as in transcriptomic analysis in various patients with PRAAS showed the induction of the transcription factor ATF3 [30,172]. Therefore, detailed studies of these proteotoxic stress axes in proteasomopathies are required to identify promising therapeutic targets. First *in vitro* experiments, targeting the ISR kinases PKR and GCN2 by chemical inhibition, reduced the type I IFN levels in patient’s cells significantly [12,61]. The serine/threonine kinase GCN2 is known for sensing amino acid deprivation in cells by its association with uncharged tRNA [173]. Proteasome dysfunction can lead to the activation of GCN2, probably due to the lack of recycled peptides within the arrested degradation process [174]. In proteasomopathies, proteasome dysfunction is often associated with the polyubiquitin-labelled protein aggregates and/or altered proteasome activity (Table 2, Table 3), resulting in a stalled peptide generation and thus limited access to amino acids in cells. Moreover, the recently identified sensor kinase PKR underscores the importance of the ISR involvement through its activation by interleukin (IL)-24 [175] and encourages ongoing research and future application of treatment alternatives, complementing the currently employed anti-interferon therapy [34,176,177]. Especially in the case of GCN2, the mechanistic understanding between its link to type IFN-I production is not fully understood. Besides its role to sense amino acid deprivation, mitochondrial dysfunction and ISR activation were observed in several studies and might indirectly connect the ISR to the type I IFN activation [178].

In addition, the involvement of metabolic cues in particular the disrupted lipid metabolism observed in PRAAS and PRNDD [11,12] underlines the critical control of metabolic pathways by targeted proteolysis through the UPS [12]. Alterations within the lipid metabolism are known for proteasomopathies; however, the link between the proteasome and a dysregulated lipid metabolism is not fully understood. An activated type I IFN signalling influences the lipid metabolism in cells, suggesting an indirect link between proteasome dysfunction and lipid metabolism [179]. Vice versa, lipotoxicity can cause dysregulation of type I IFN signalling [20,180].

## CONCLUSION AND FUTURE PERSPECTIVES

**Figure 5:**
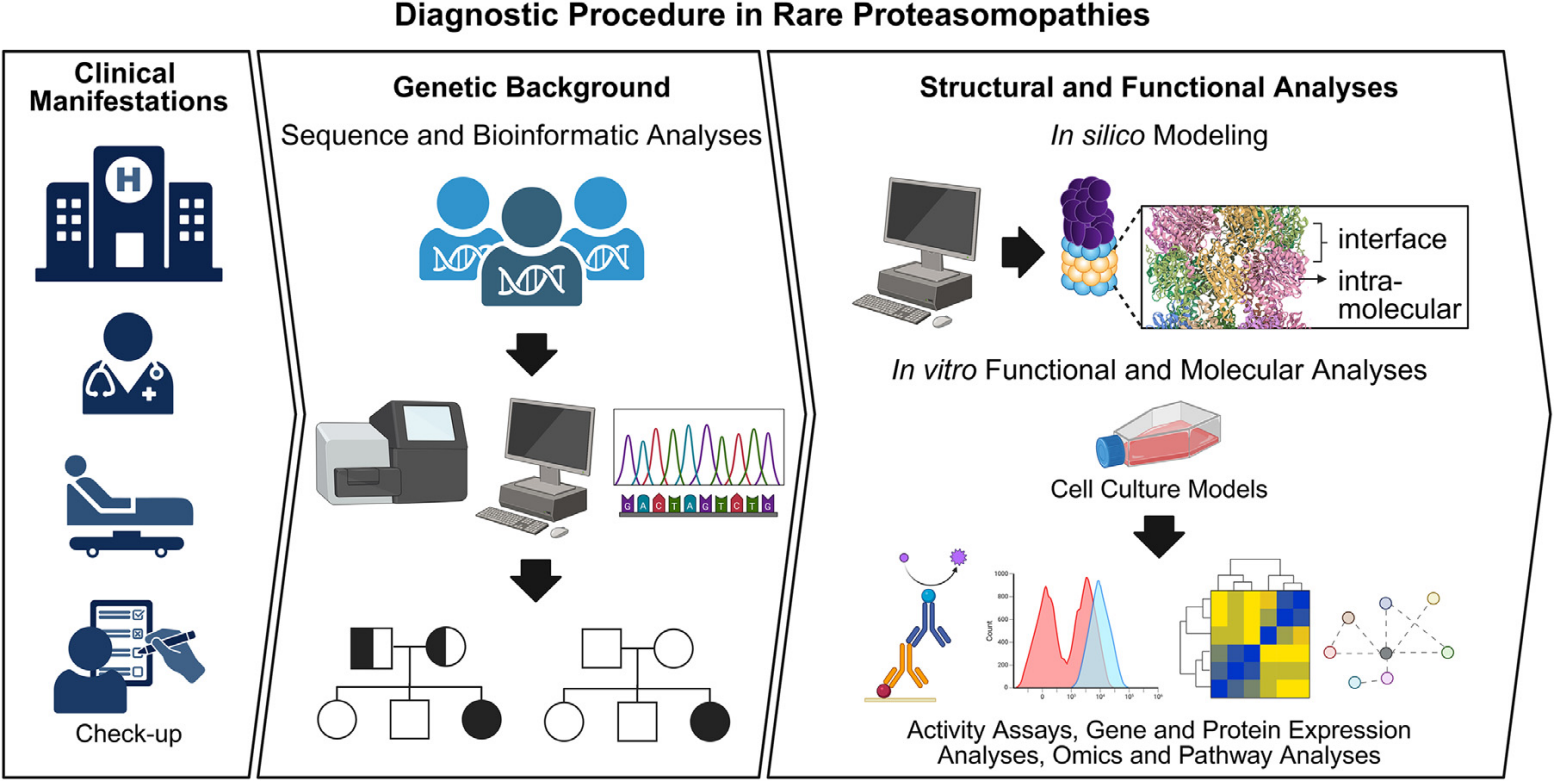
Illustration of diagnosis in rare proteasomopathies. Analysis of proteasome variants encompasses its clinical manifestations, the genetic unravelling of the variant, which further drives the structural and functional analysis of the variants. Several cell culture models strengthen the tissue- and cell-specific studies upon proteasome dysfunction. The proteasome structure is derived from PDB: 8QYO. Created in BioRender. Venz, S. (2025) https://BioRender.com/tincwdn.

Our understanding of the molecular details causing rare proteasomopathies has been considerably increased. The ability of variant-specific structural modelling with AI-based AlphaFold3-technology and improving experimentally obtained structures revolutionised the interpretation of structural consequences (Fig 5). Furthermore, this approach, in combination with genetic prediction tools [[181], [182], [183]], considerably improves the prediction of pathogenicity of single variants without the need for experimental approaches and accelerates diagnosis. Thus, establishing this accurate prediction tool for diagnostics in proteasomopathies, but also other rare diseases has the potential to support early diagnosis. The development of panels with additional biomarkers, such as protein aggregates, may also help in diagnostics of proteasomopathies in the future.

While structural modelling allows the prediction of dysfunctional conformational changes to near atomic resolution, there is an urgent need to advance our knowledge of cell-specific consequences of proteasome variants and the dysregulated signalling pathways behind. Future assessment of proteasome variants should consider a standardised molecular analysis of cell-specific pathways to understand the differences between PRAAS and PRNDD or to identify signalling hubs as therapeutic targets. Induced pluripotent stem cells and organoids can serve as suitable platform for comprehensive studies of the early developmental stages of various cell types to elucidate early consequences of proteasome dysfunctions in differentiation processes [184]. Even if this would improve the diagnostic procedures in proteasomopathies, several questions remain elusive: Are there distinct molecular patterns or regulators that drive the clinical phenotype into either PRAAS or PRNDD? Is there another sensor, besides PKR and GCN2, which may explain the link between proteotoxic stress responses and the type I IFN signalling? Are the type II and type III IFN signalling pathways also involved and at what stage or hierarchy? What is the tissue-specific impact of proteasome variants and to what extent do tissue-specific effects occur during embryonic development? Are there rescue mechanisms that ensure the survival of dominant-negative proteasome LOF variants in early development? How is the distribution of proteasome isoforms in tissues, and how are they established by specific expression patterns or biogenesis pathways?

## CRediT authorship contribution statement

**Sophie Wolfgramm:** Writing – review & editing, Writing – original draft, Visualization, Investigation, Conceptualization. **Flavia Llorente Alvarez:** Writing – review & editing, Writing – original draft, Investigation, Conceptualization. **Franziska G Thiel:** Writing – review & editing. **Martin Wendlandt:** Writing – review & editing. **Elke Krüger:** Writing – review & editing, Funding acquisition, Conceptualization.
